# Local Adaptation to Current but Not Future Climate in Seasonally Dry Tropical Forests: Population Genomic Evidence in a Neotropical Legume Tree

**DOI:** 10.1111/eva.70302

**Published:** 2026-08-07

**Authors:** Francisco J. Velásquez‐Puentes, Walter Durka, Colin Hughes, R. Toby Pennington, Jens J. Ringelberg, Renske E. Onstein, Christopher D. Barratt

**Affiliations:** ^1^ Universidad del Norte Barranquilla Colombia; ^2^ Leipzig University Leipzig Germany; ^3^ German Centre for Integrative Biodiversity Research (iDiv) Halle‐Jena‐Leipzig Leipzig Germany; ^4^ Department of Community Ecology (BZF) Helmholtz Centre for Environmental Research – UFZ Halle Germany; ^5^ Department of Systematic & Evolutionary Botany University of Zurich Zurich Switzerland; ^6^ Department of Biology Washington University St. Louis Missouri USA; ^7^ Department of Geography University of Exeter Exeter UK; ^8^ Missouri Botanical Garden St. Louis Missouri USA; ^9^ Biosystematics Group Wageningen University and Research Wageningen the Netherlands; ^10^ Naturalis Biodiversity Center Leiden the Netherlands; ^11^ Institute of Biology Leiden (IBL), Leiden University Leiden the Netherlands; ^12^ Animal Breeding and Genomics Wageningen University and Research Wageningen the Netherlands

## Abstract

Neotropical seasonally dry biomes are amongst the world's most threatened ecosystems and are predicted to lose more biodiversity with climate change. The capacity of natural populations to respond to these changes depends on their genetic variation, but is poorly understood in most Neotropical seasonally dry forest species. Here, we used genome‐wide single nucleotide polymorphism (SNP) data for 109 individuals across 12 Colombian populations of *Enterolobium cyclocarpum* , a widespread deciduous legume tree found in seasonally dry tropical forests, human‐disturbed and open landscapes from Central and Northern South America, to (1) explore population structure across the landscape, (2) determine local adaptation to heat and drought stress, and (3) assess genomic offset and adaptive potential under future climate change. Our results suggest clear genomic differentiation among regions and populations, as well as an uneven spatial distribution of adaptive alleles associated with drought and heat stress, together suggesting different degrees of local adaptation to climate across the landscape. Furthermore, all regions investigated (i.e., Caribbean, inter‐Andean valleys, and Orinoquía) showed limited adaptive potential under future climate change scenarios. For some regions, gene flow may help buffer the effects of environmental change by bringing in adaptive alleles, however, this will likely be insufficient to counteract predicted mal‐adaptation in certain regions, such as in the Orinoquía. Our results suggest that both barriers to gene flow (e.g., orography) and varying heat and drought stress conditions have shaped the genomic composition and adaptive potential of the species. *Enterolobium cyclocarpum* populations have adapted locally to current climate, but this adaptation may not be sufficient to cope with future climate change, particularly where population connectivity is low. Our findings have relevance for the conservation of species in highly threatened Neotropical biomes, such as seasonally dry tropical forests, which are expected to experience rising temperatures and greater drought under future climate change.

## 1 Introduction

Neotropical landscapes exhibit high climatic and topographic heterogeneity (Hughes et al. 2013), which has contributed to their exceptional species and genetic diversity (Antonelli and Sanmartín 2011; Miraldo et al. 2016; Hazzi et al. 2018; Tietje et al. 2023). Among Neotropical biomes, seasonally dry forests and savannas, characterized by seasonal drought, are undergoing severe transformation due to habitat transformation (Pennington et al. 2018). Seasonally dry tropical forests comprise the most endangered tropical biome globally (Janzen 1988), being highly fragmented (Pennington et al. 2018) and having less than 10% of their original cover remaining intact in many Central and South American countries. Fragmentation within Neotropical Seasonally Dry Forests (NSDFs) also occurs naturally, with disjunct floristic nuclei scattered across the Neotropics (Pennington et al. 2009; DRYFLOR et al. 2016). This disjunct distribution is thought to have persisted through time, implying limited historical dispersal between isolated floristic nuclei (Pennington et al. 2009). NSDFs have a deep evolutionary history, with some of the oldest plant lineages from this biome dating back to the middle to late Eocene (c. 47.3–33.9 Ma) (Martínez et al. 2021). In contrast, savannas are thought to be younger, with many of their woody lineages emerging after transitions from humid forests and other biomes over the past 8–5 Myr in the Neogene (Simon et al. 2009; Jaramillo 2023).

Current global change trends, including intense, prolonged, and often unpredictable periods of warming and drought, are likely to accelerate the degradation, fragmentation, and desertification of dry biomes (Bastin et al. 2017), including NSDFs. Consequently, there is an increasing need to understand how seasonally dry tropical biomes generally will respond to global climate trends. Trees are sessile organisms with usually long generation times, which make them more vulnerable to climate change (Aitken et al. 2008; Holliday et al. 2017). More frequent and severe drought events have already driven widespread tree mortality worldwide and are expected to intensify in the future (Allen et al. 2010; Greenwood et al. 2017; Brodribb et al. 2020; Hartmann et al. 2022). Understanding how trees cope with and adapt to warming and drought is critical for predicting the fate of biodiversity under global change, particularly in endangered tropical biomes such as NSDFs (Lindenmayer and Laurance 2016; Menezes‐Silva et al. 2019; Doughty et al. 2023).

Understanding how trees locally adapt can be determined by estimating the adaptive genetic potential of populations and species, providing data for informing conservation strategies (Alberto et al. 2013; Capblancq et al. 2020; Whiting et al. 2024). However, assessing adaptive potential remains challenging due to multiple factors shaping genetic variation, such as geographic isolation, both topographic and by distance, climatic and ecological differences, and demographic history. These factors drive population divergence (Hazzi et al. 2018; Pérez‐Escobar et al. 2022) through both adaptive (i.e., natural selection) and neutral (i.e., mutations and genetic drift) processes, which often interact synergistically (Nosil et al. 2009). In addition, other sources of genetic variation such as structural variants (Wellenreuther et al. 2019) can play a role in adaptation. Moreover, spatial and temporal variation in population isolation, selection and gene flow create heterogeneous patterns in the adaptive component of genetic variation (Nosil et al. 2009; Orsini et al. 2013), which is crucial for species survival (Chung et al. 2023). However, the mechanisms driving neutral and adaptive genetic variation remain relatively under‐studied in Neotropical organisms, particularly in threatened biomes such as NSDFs. Most studies have focused on macroevolutionary patterns across NDSFs as a whole (DRYFLOR et al. 2016) and especially across the dry diagonal, the Caatinga and Cerrado (Collevatti et al. 2019, 2020; Vieira et al. 2022), whereas studies from NSDFs in central America and northern South America remain scarce.

Advances in high throughput sequencing allow genotyping thousands to millions of genome‐wide Single Nucleotide Polymorphisms (SNPs), providing the resolution needed to reveal complex neutral genetic structure and genetic diversity patterns, as well as detecting signals of gene flow and local adaptation. Leveraging large SNP datasets, Genotype‐Environment Association (GEA) methods in particular have proven reliable for detecting potential candidate genomic regions involved in local adaptation (Rellstab et al. 2015; Hoban et al. 2016; Capblancq and Forester 2021), which can be assessed in different populations across the landscape. Quantifying the spatial distribution of these adaptive regions (i.e., adaptive alleles associated to a given SNP genotype, hereafter “adaptive potential”) across populations provide an important guide to assess the capacity of populations to cope with future climate change (Razgour et al. 2019; Capblancq et al. 2020; Waldvogel et al. 2020; Dauphin et al. 2023). Furthermore, genomic offset approaches, based on modeled allele‐climate relationships (Capblancq et al. 2020; Gougherty et al. 2021; Rellstab et al. 2021), allow the quantification of mismatches between current and predicted adaptive allelic frequencies necessary to cope with future climatic conditions (i.e., “genetic mal‐adaptation”). This approach is particularly relevant for long‐lived organisms, such as trees, where the threat of climate change may occur within the lifespan of individuals (Capblancq et al. 2020). For instance, genomic offsets have been used to assess genetic mal‐adaptation to future environmental conditions in *Dalbergia cochinchinensis* and *Dalbergia oliveri* (Fabaceae) from tropical forests in the Greater Mekong in Southeast Asia (Hung et al. 2023), *Quercus acutissima* (Fagaceae) (Yuan et al. 2023) and *Populus koreana* (Salicaceae) (Sang et al. 2022) from temperate deciduous forests in China, *Quercus rugosa* (Fagaceae) from temperate zones in Mexico (Martins et al. 2018), and *Populus balsamifera* (Salicaceae) from boreal forests in North America (Fitzpatrick and Keller 2015). However, in the Neotropics, relatively few studies have examined the adaptive potential and genetic mal‐adaptation in tree populations.

Here, we address this knowledge gap by investigating the mechanisms shaping the genetic structure, adaptive potential and genetic mal‐adaptation in a Neotropical tree species distributed across seasonally dry tropical forests throughout Colombia. Specifically, we ask the following questions: (1) how are populations genetically structured across the landscape? We hypothesize that both isolation by distance and isolation by topographic barriers (e.g., Andes) shape population genetic structure. Hence, we expect genetic distance to increase with both geographical and environmental distance (H1). (2) Which SNPs show genotype‐environmental associations with high temperature (heat stress) and low precipitation (drought stress), and how do their allele frequencies and spatial distribution vary across its range? We hypothesize that drought and heat stress impose differential selective pressures and are associated with adaptive alleles. We expect heat and drought intensity to vary across the species' range and hence detect variation in local adaptation across populations (H2). (3) Which populations and regions are most mal‐adapted under future heat and drought stress predictions? We hypothesize that populations with few adaptive alleles and limited gene flow will show high genomic offsets and face greater risk under future climate conditions due to potential mal‐adaptation and low spatial and genetic connectivity (H3).

## 2 Methods

### 2.1 Study System

**FIGURE 1:**
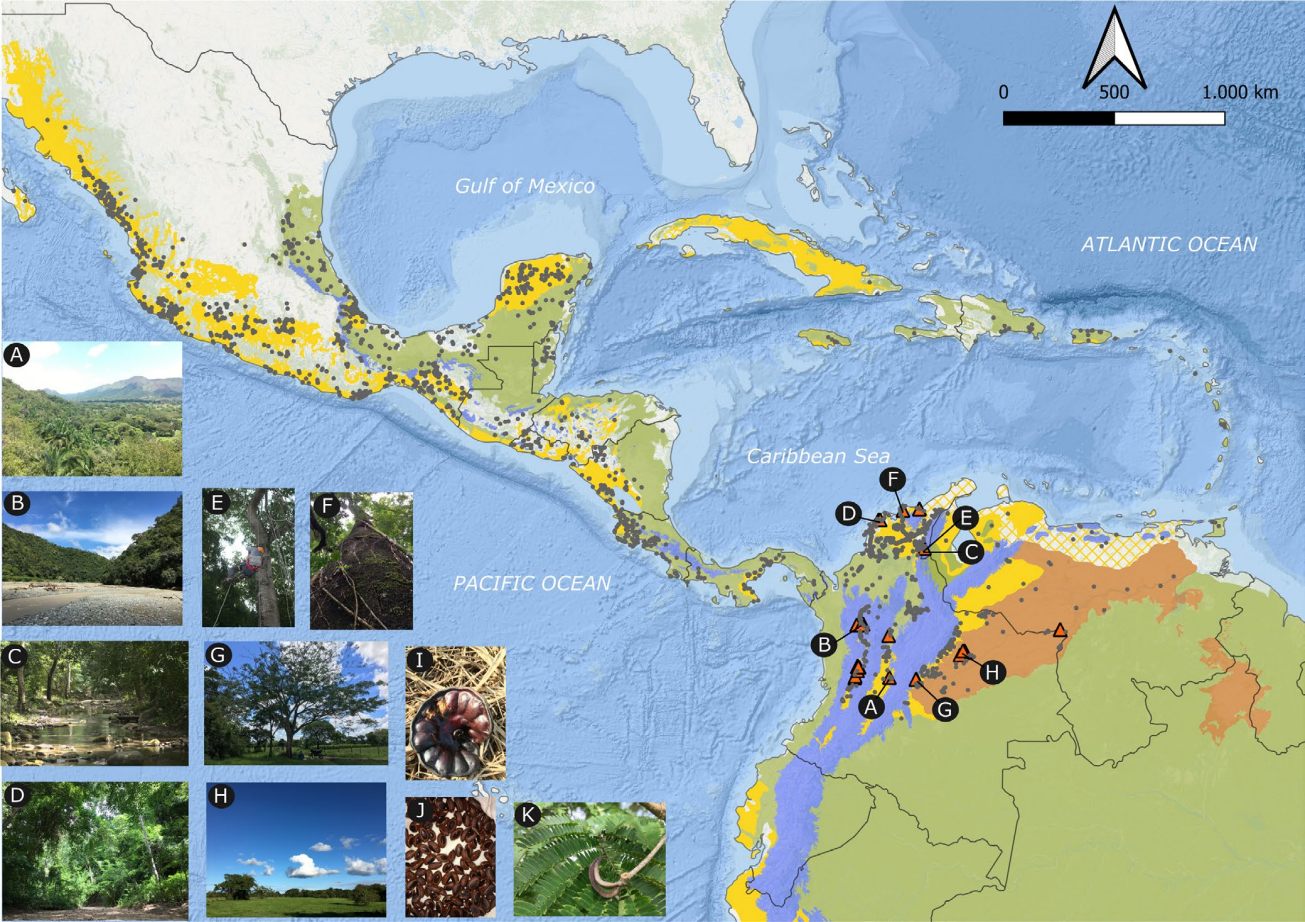
Distribution of *Enterolobium cyclocarpum* across south‐central Mexico, Central America and northern South America, and sampling sites. Gray dots indicate individual occurrences (based on validated records from the Global Biodiversity Information Facility), and red triangles show the sampling localities. (A) Seasonally dry tropical forest landscape from the Upper Magdalena valley, (B) Seasonally dry tropical forest in the northern Cauca Valley, (C and D) Riparian habitats where *E. cyclocarpum* is commonly found in seasonally dry tropical forests. (E and F) Tall individuals sampled from the Caribbean region, (G) A short individual sampled in an agroforestry landscape in the Orinoquía, (H) Human‐modified savanna used for cattle ranching in the Orinoquía, (I–K) Pod, seeds and leaves of *E. cyclocarpum* . Shaded colors indicate major biomes (following Olson et al. 2001): Blue = Andes, green = tropical wet forest, yellow = seasonally dry tropical forest, brown = savanna, yellow cross‐hatch = xeric/shrubland.

*Enterolobium cyclocarpum* (Jacq.) Griseb. (Leguminosae; subfamily Caesalpinioideae; tribe Mimoseae) is a widespread mimosoid legume tree found most frequently in lowland deciduous and semi‐deciduous, seasonally dry tropical forests from central Mexico south through Central America and northern South America (Figure 1) (Record and Hess 1943). Although more common in seasonally dry tropical forests, the species tolerates a broad range of moisture regimes and is also found in tropical moist and premontane moist forest zones (Hunter 1989). The species is commonly referred to as the ear pod tree or elephant ear tree in reference to its large, brown, indehiscent, ear‐shaped pods (Figure 1I). These fruits represent an iconic Neotropical evolutionary anachronism dependent in the past on endozoochorous seed dispersal by now extinct megafauna (e.g., Pleistocene horses, gomphotheres, or giant ground sloths) (Janzen and Martin 1982), and nowadays on horses and cattle in agroforestry landscapes (Janzen 1981b, 1982), as well as by howler monkeys (Rockwood and Glander 1979; Cuervo‐Díaz et al. 1986) and tapirs (Janzen 1981a, 1981c) in more intact forests. *Enterolobium cyclocarpum* is shade intolerant (Hatheway and Baker 1970), exhibits rapid growth, and has deciduous bipinnate compound leaves which are shed during the dry season, with new leaves emerging shortly before the rainy season, coinciding with the blooming of globose inflorescences of small white flowers (Janzen 1983). In seasonally dry tropical forests, *E. cyclocarpum* trees are most abundant in areas of frequent local disturbance alongside or close to water, in swamps, and river margins (Janzen 1983; Hunter 1989). The species is also commonly found in fragmented populations in human‐modified landscapes such as cattle ranches and agricultural fields where the species provides shade in pastures and the fruits provide valuable livestock feed (Janzen 1981c; Hunter 1989). The species has a diploid genome (2n = 26) (Atchison 1951), making it a suitable candidate for bioinformatic processing of double‐digest‐restriction‐site associated DNA sequencing (ddRADseq) data (Catchen et al. 2013). The combination of a diploid genome, the widespread and disjunct distribution, long‐generation times, and presence along climatic gradients in heterogenous landscapes—such as those across deciduous and semi‐deciduous lowland forests in Colombia that are intersected by the Andes—make *E. cyclocarpum* an ideal candidate for studying the adaptive potential to climate change in seasonally dry tropical forest trees.

### 2.2 Data and Sampling

**FIGURE 2:**
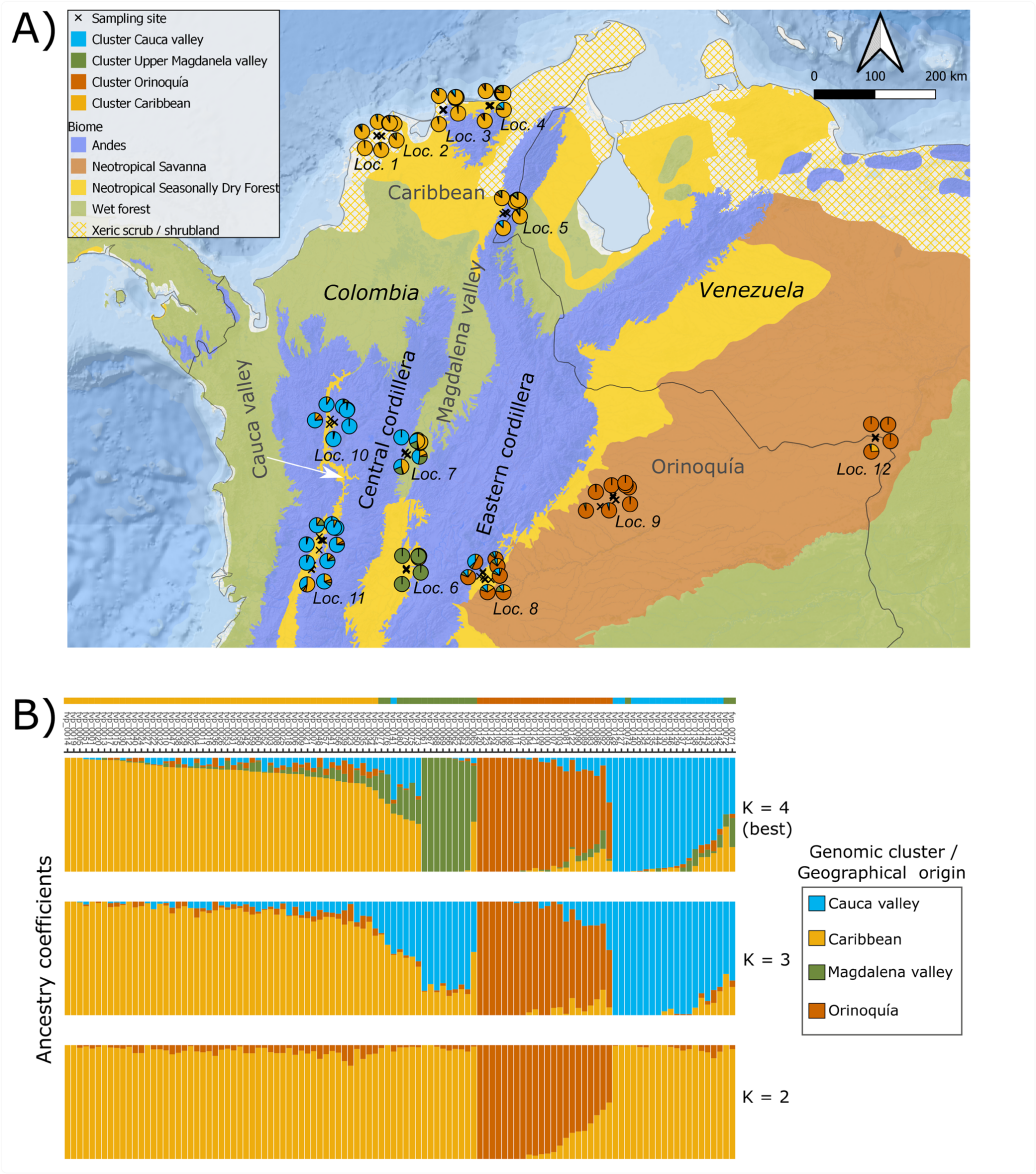
Geographic structure of *Enterolobium cyclocarpum* populations across Colombia. (A) Distribution of genetic clusters (k = 4) depicted in pie charts across the landscape positioned around the true location of the individual samples, labeled with an “x”. (B) Proportions of ancestry of each individual, assigned to inferred genetic clusters for K = 2, 3 and 4. Colors represent genetic clusters corresponding to a specific geographical region. The white arrow in A indicates the Cauca Valley. Code names in B above the bars represent sample identifiers (see Tables S1 and S2). The colored boxes above sample names indicate the geographic region where the samples were collected in the field.

We collected leaf samples from 118 individuals of *E. cyclocarpum* in the field in seasonally dry tropical forests, semi‐deciduous forests and moist zones from Colombia, covering the southern part of its distribution range in the Neotropics (Figure 2). Sampling covered 12 localities spanning a temperature and precipitation gradient (Table S1), including seasonally dry tropical forests of the Caribbean region of Colombia, seasonally dry tropical forests of the inter‐Andean valleys (north and south of the Cauca valley, and Upper Magdalena valley), moist forests of the Middle Magdalena valley, and seasonally dry tropical forests and riparian forest in the Orinoquía (from the piedmont to the Colombia‐Venezuela border) (Figure 1A; Tables S1 and S2). Sampling sites exhibited varying degrees of human disturbance ranging from no transformation to secondary forests and cattle ranches in agricultural landscapes. The primary drivers of landscape alteration in these areas were agricultural and cattle ranch expansion. Populations located in the Caribbean are found in the most intact seasonally dry tropical forest patches and represent the tallest trees among all individuals sampled (Figure 1). On the other hand, populations from the Orinoquía were found in the most open landscapes. Given the extent of habitat modification, human‐induced transformation may influence local genetic variation and local adaptation. Although we did not explicitly control for this factor, we minimized its impact by sampling mostly large adult trees, excluding younger individuals or treelets. These younger trees are more likely to come from human‐mediated transplanting and cattle translocation in transformed landscapes.

### 2.3 Climatic Data and Climate Change Scenarios

For all analyses we selected climate variables associated with drought and heat stress, which are thought to be important physiological constraints for tree species in lowland tropical forests (Allen et al. 2010). Specifically, we chose precipitation of the driest month (PDM), average monthly precipitation during the warmest quarter of the year (PWQ), temperature seasonality (TS), and average monthly mean temperature over the driest quarter of the year (MTDQ), which showed the lowest collinearity (i.e., Variance Inflation Factor [VIF] values ranging between 1.2 and 2.2. See also pairwise Pearson correlations in Figure S1). Climate variables were extracted from the CHELSA version 2.1 climate data portal (Karger et al. 2017) for the period 1981–2010 at a resolution of 1 km, based on individual geographic coordinates taken in the field (Table S1). We generated different climate change scenarios using the chelsa cmip6 python module (Karger et al. 2023), accessed via the reticulate R package (Ushey et al. 2023). This module provides climate projections based on the Coupled Model Intercomparison Project Phase 6 (CMIP6) (O'Neill et al. 2016), using CHELSA version 2.1 (Karger et al. 2017) as a baseline. We selected the period 1981–2010 as the baseline and projected climate data to three different periods (2041–2060, 2061–2080 and 2071–2100) under two global development scenarios (Fossil‐fueled development Shared Socioeconomic Pathway 5–8.5 [SSP5‐8.5] and 2–4.5 [SSP 2–4.5] emission models), and three different global climate circulation models (GFDL‐CM4 [Geophysical Fluid Dynamics Laboratory's CM4.0 physical climate model; Held et al. 2019], MPI‐ESM‐1.2‐LR [Max Planck Institute for Meteorology Earth System Model version 1.2—low resolution; Mauritsen et al. 2019], and EC‐EARTH‐3‐VEG‐LR [Earth Consortium Version 3—Vegetation—low resolution; Döscher et al. 2022]). All selected climate models simulate atmosphere–ocean interactions and large‐scale circulation patterns, working well for pressures and ocean patterns that control ENSO (El Niño Southern Oscillation) and NASH (North Atlantic Subtropical High), making climate predictions for northern South America more reliable (Arias et al. 2021, 2025). EC‐Earth3‐Veg‐LR additionally incorporates dynamic vegetation processes, including vegetation‐climate feedbacks (e.g., evapotranspiration, carbon cycling, and albedo changes; Döscher et al. 2022). Climate data were stored as raster layers for both present and future periods, across models and scenarios, for each variable. These rasters covered the main areas of distribution of the species in northern South America spanning the Caribbean, inter‐Andean valleys, and the Orinoquía in Colombia and Venezuela (longitude = −87° to −67°, latitude = 1°–12.4°). To improve computational efficiency, climate model rasters were downscaled by a factor of 10 by averaging groups of 10 × 10 cells using the aggregate function in the raster R package (Hijmans 2023). Climatic predictors were extracted using centroids of each sampling locality and were used to predict genomic offsets under the different climate change scenarios.

### 2.4 High‐Throughput Sequence Data and Filtering

Our study deployed double digest restriction‐site associated DNA (ddRAD) sequencing to generate libraries representing a subset of the genome (Peterson et al. 2012). We extracted genomic DNA using the DNA extraction Plant Kit Qiagen, double‐digested the DNA and followed the library preparation protocol of Durka et al. (2025). Multiplexed samples were sequenced paired‐end. After sequencing, we demultiplexed the reads using the command process_radtags from the Stacks 2.0 pipeline (Rochette et al. 2019). We recovered raw genomic sequences for 115 samples and retained 109 samples for analysis after post‐processing of raw sequences. Raw DNA sequences were assembled de novo (i.e., without mapping to a reference genome, which is not available for *E. cyclocarpum* ) using Stacks 2 (Rochette et al. 2019). To optimize the de novo locus assembly and calling of SNPs, we followed established practices (Paris et al. 2017), which maximize the biologically relevant information contained within the sequence data (i.e., by maximizing polymorphisms in the data while minimizing “false” RAD loci caused by over‐ or under‐merging). Specifically, we aimed to determine the most effective combination of parameters: m (minimum number of raw reads required to form a stack or putative allele), M (the allowed number of mismatches between putative alleles to define putative loci), and *n* (the allowed number of matches between putative loci during catalog construction). To achieve this, we set M equal to *n* at 4 and ran Stacks with m values ranging from 1 to 9 to identify the optimal parameter combination for our dataset that maximized the number of assembled sites and polymorphic loci, with the aim of achieving 80% polymorphic loci (i.e., r80). The ideal combination for our dataset was determined to be M = n = m = 4.

Following de novo assembly, the data were further filtered using VCFtools version 0.1.16 (Danecek et al. 2011) by removing indel sites (i.e., − remove‐indels), allowing sites with a maximum of 50% missing data (i.e., − max‐missing 0.5), including only bi‐allelic sites (i.e., − min‐alleles 2 and – max‐alleles 2), sites with mean read depths between 5 and 100 (i.e., − min‐meanDP 5, and – max‐meanDP 100), and genotypes with mean read depths between 5 and 100 (i.e., − minDP 5, and – maxDP 100). Additionally, we filtered the data by setting up the minimum percentage of individuals in a population required to process a locus equal to 20% (i.e., − r 0.2) and restricted data to only a single SNP per locus (i.e., −write‐single‐snp). We did not restrict sites by minimum minor allele frequencies, nor by the maximum level of heterozygosity a variable site can possess to be included, to recover low‐frequency sites under selection (Ahrens et al. 2018). The resulting dataset comprised 109 individuals genotyped at a total of 29,266 SNPs. This dataset exhibited a 28.62% rate of missing data, which were subsequently imputed by assigning the most common genotype for each locus (represented by a single SNP) across individuals for further analyses.

### 2.5 Population Structure and Modes of Isolation Across the Landscape

To examine the genetic population structure of *E. cyclocarpum* and assess whether environmental and/or geographic distance influenced population differentiation (H1), we first used sparse‐non‐negative matrix factorization (SNMF), following Frichot et al. (2014). We determined the optimal number of genetic clusters (i.e., K) and ancestry coefficients per individual employing the snmf function from the R package LEA (Frichot and François 2015). We explored values of K ranging from 1 to 10, using an entropy criterion and cross‐validation technique as implemented in the function, selecting K based on the inflection point from the plotted cross‐entropy values. To assess the impact of geographical factors, specifically distance and environment (i.e., Andes) on genetic structure, we calculated genetic pairwise distances between unique sampling localities using Weir and Cockerham's *F*_ST_ (Weir and Cockerham 1984) via the hierfstat package in R (Goudet 2005). *F*_ST_ values were calculated using three different sets of SNPs: the complete dataset, a subset of candidate SNPs (detailed in the section below), and a third set of SNPs associated with neutral alleles (see Supporting Information for details) to better understand how geographic and environmental distances shaped the distribution of adaptive versus neutral alleles. Comparisons were then made between *F*_ST_/(1 − *F*_ST_) (Rousset 1997) estimates, geodesic distance, and environmental distance. To calculate environmental distance, we first constrained population connectivity between sampled localities by elevation, reflecting *E. cyclocarpum* 's restriction to lowland tropical forests. This involved modeling connectivity as a decreasing function of elevation, such that higher elevations corresponded to reduced connectivity. To implement this, we used a raster of elevation data for Colombia and computed a transition matrix across the landscape connecting grids based on the elevation criteria using the R package gdistance (Van Etten 2017). We also restricted connectivity to areas below 1600 m, the species' maximum recorded elevation in Colombia (Salazar‐Giraldo et al. 2021). Environmental distance was then quantified across the elevation‐based transition matrix using the function costDistance from the gdistance package. Geodesic distance was estimated using the function geodist from the geodist R package (Padgham and Sumner 2021). Both distances were calculated between population centroids and compared with genetic distances estimated from different SNP datasets (i.e., all SNPs, candidate SNPs, and SNPs associated with neutral alleles) using Mantel tests implementing the function mantel from the R package vegan (Oksanen 2010). Correlations were estimated using Pearson's correlation coefficient, and significance was assessed with 9999 permutations. Additional Mantel tests were performed within‐ and between‐region population pairs using the complete SNP dataset to evaluate the effects of isolation by distance and the environment among specific populations (i.e., those located on the same Andean flank or on opposite sides).

### 2.6 Identification of Adaptive Alleles at Candidate SNPs

Throughout this study, the term “adaptive” refers to putatively adaptive alleles at candidate SNPs showing genotype‐environment associations. To identify SNPs showing genotype‐environment associations, we performed a partial redundancy analysis (RDA), a constrained ordination method shown to minimize false positive rates under various demographic scenarios and sampling designs (Forester et al. 2018). The full genotype data matrix (i.e., for all 109 individuals) was used as the response variable, along with the four climatic predictors (Table S1). Analyses were conducted using the rda function in the vegan package in R (Oksanen 2010), with latitude, longitude, and the first two principal components from a principal component analysis (PCA) of the genotype data included as covariates to account for geographic and population structure. As variables do not have the same units, the scale parameter was set to true, so all variables have equal weight when performing the partial RDA. SNPs showing genotype‐environment associations (hereafter “candidate SNPs”) were defined as those with loadings ±2.8 standard deviations from the mean on any of the four RDA axes, representing a conservative threshold (Forester et al. 2018). We also detected 3 duplicate candidate SNPs occurring on multiple RDA axes and removed them, as a clear association with a single environmental variable was absent. Each genotype is coded as 0, 1 or 2, according to the dosage of the alternative allele (i.e., with 0 indicating homozygosity for the reference allele, 1 heterozygosity, and 2 homozygosity for the alternate allele), and was associated with the environmental variable showing the strongest correlation based on the allele dosage (see File S1). Positive correlations indicate that the alternative allele of a genotype may be associated with increasing temperature (i.e., warmer climates imposing heat stress) or precipitation (i.e., wetter conditions) suggesting it contributes to adaptation to warmer or wetter conditions. Conversely, negative correlations indicate the alternative allele may be associated with decreasing temperature or precipitation, suggesting it contributes to adaptation to cooler or drier conditions.

### 2.7 Local Adaptation Along Climatic Gradients

To explore the distribution of candidate SNPs across precipitation and temperature gradients and determine whether adaptive alleles exhibited clinal patterns associated with increasing heat and drought stress (H2), we examined both the per‐individual representation of candidate SNPs carrying adaptive alleles and population‐level allele frequencies along climatic gradients. We quantified the number of candidate SNPs carrying at least one adaptive allele for each individual (note that individuals may carry zero, one or a maximum of two alternate/reference alleles associated with each candidate SNP) and examined how the per‐individual proportion of candidate SNPs carrying adaptive alleles varied across climatic gradients. In addition, we examined population allele frequencies for each candidate SNP along climatic gradients to assess adaptive variation at the population scale. We used non‐linear least squares regression, implemented with the nls function in the R package stats (R Core Team 2022) to model the per‐individual proportion of SNPs carrying adaptive alleles as a function of the corresponding standardized climatic variable. Climatic variables were standardized by centering each value to its mean and scaling by its standard deviation using the scale function in R (R Core Team 2022). Specifically, we fitted a sigmoidal cline using the hyperbolic tangent function (Szymura and Barton 1986) using the equation *y = a* + (*b* − *a*) · tanh[(*x* − *c*)/*d*], where *a* and *b* represent the lower and upper frequency limits in the *y*‐axis, *c* is the center of the cline along the *x*‐axis, *d* the cline width, and *x* the scaled environmental value. The parameter *a* shifts the curve vertically, and (*b − a*) determines the amplitude. Model fit was assessed using the *r*‐squared statistic. Statistical significance of model parameters was assessed using *p*‐values reported by the summary function from the R package stats, based on approximate *t*‐statistics derived from a linear approximation of the nonlinear model. Subsequently, we explored the allele frequencies of each candidate SNP across climatic gradients using generalized additive models (GAMs) implemented with the gam function in the R package gam (Hastie 2023).

### 2.8 Genomic Offsets and Gene Flow Across the Landscape

We identified populations and regions with the highest and lowest potential genomic mal‐adaptation under projected future heat and drought stress scenarios by calculating genomic offsets (H3). To do so, we modeled the relationship between allele frequencies of adaptive alleles at candidate SNPs, as well as neutral alleles, and the selected climatic variables (Table S1) using Gradient Forests models (Fitzpatrick et al. 2021) implemented with the gradientForest function from the R package gradientForest (Ellis et al. 2012). These were used first with allele frequencies from a set of candidate SNPs showing positive associations between the alternative allele and increasing temperature (i.e., warmer conditions) and negative associations with precipitation (i.e., drier conditions), and a second set including all candidate SNPs recovered in the partial RDA. In addition, we further estimated genomic offsets using sets of SNPs associated with neutral alleles matching the number of the two sets of candidate SNPs to test whether the observed offsets exceeded patterns expected under neutral allele‐frequency change (see Supporting Information for details). Genomic offsets are estimated as the expected allele frequency mismatch under future climate, computed as Euclidean distances between modeled present‐day allele frequencies, and those predicted to be adaptive under future climate change based on the Gradient Forest model (Fitzpatrick and Keller 2015). Genomic offset maps were then generated for major regions of Colombia—the Caribbean, inter‐Andean valleys and Orinoquía—by calculating genomic offsets and assessing adaptive mismatches under future (2041–2060, 2041–2060, and 2071–2100) climate projections integrating each climatic predictor according to the SSP5‐8.5 and SSP2‐4.5 emission scenarios and the three global climate models (GFDL‐CM4, MPI‐ESM‐1.2‐LR and EC‐EARTH‐3‐VEG‐LR).

As a complementary analysis, we assessed gene flow among populations to evaluate whether populations with high genomic offsets (i.e., greater risk of future genomic mal‐adaptation based on future climate projections compared with contemporary gene–environment associations) were also vulnerable due to limited population connectivity. We estimated directional relative migration rates (hereafter “relative gene flow”) between population pairs using the divMigrate function from the R package diveRsity (Keenan et al. 2013; Sundqvist et al. 2016). Relative gene flow ranges between 0 and 1 and was estimated from the number of migrants per generation (i.e., Nm; Alcala et al. 2014), scaled by the maximum value across all pairwise population comparisons, each comprising bi‐directional estimates (e.g., from population i to j and from j to i). Nm (Alcala et al. 2014) is calculated as (1/4)[(1 − *G*_ST_)/*G*_ST_] × [(*n*−1)/*n*]^(2)^ × [(1 − *D*)/(1 – *D* × {(*n* − 1)/*n*})], where *G*_ST_ (Nei and Chesser 1983) and *D* (Jost 2008) are indices of genetic differentiation (based on allele fixation and allele composition, respectively), and *n* is the number of populations. Relative gene flow should be interpreted as a comparative measure of connectivity (i.e., reflecting gene flow strength relative to the maximum observed in the study system), rather than as absolute migration rates. We used the full SNP dataset (29,266 SNPs) to calculate relative gene flow and classified the estimated values into four categories: low (0–0.25), low to intermediate (0.25–0.5), intermediate to high (0.5–0.75), and high (0.75–1), enabling spatial comparisons of relative gene flow across the landscape.

## 3 Results

### 3.1 Population Structure

Our results reveal four major population clusters (K = 4; best‐supported K; Figure 2 and Figure S2) coinciding with four of the main geographic regions in Colombia, partially isolated by the Andean cordilleras: (i) Caribbean (i.e., Northern Colombia); (ii) Orinoquía (i.e., Eastern Colombia and borders with Venezuela); (iii) the Cauca valley (i.e., the inter‐Andean valley between the central and western cordilleras); (iv) the Upper Magdalena valley (i.e., the upper inter‐Andean valley between the central and eastern cordilleras) (Figure 2). Sampled localities in the Middle Magdalena valley show significant admixture of Caribbean, Cauca valley and Upper Magdalena valley genotypes across all individuals (Figure 2). The range of K values inferred (K = 2–4) reflect hierarchical genetic structure (Meirmans 2015) (Figure 2B). At K = 2, Orinoquía populations are clearly differentiated, forming a distinct genetic cluster, whereas Caribbean and inter‐Andean populations cluster together. At K = 3, Caribbean and inter‐Andean populations are differentiated as distinct genetic clusters. At K = 4, a fourth cluster corresponding to the upper Magdalena valley emerges.

**FIGURE 3:**
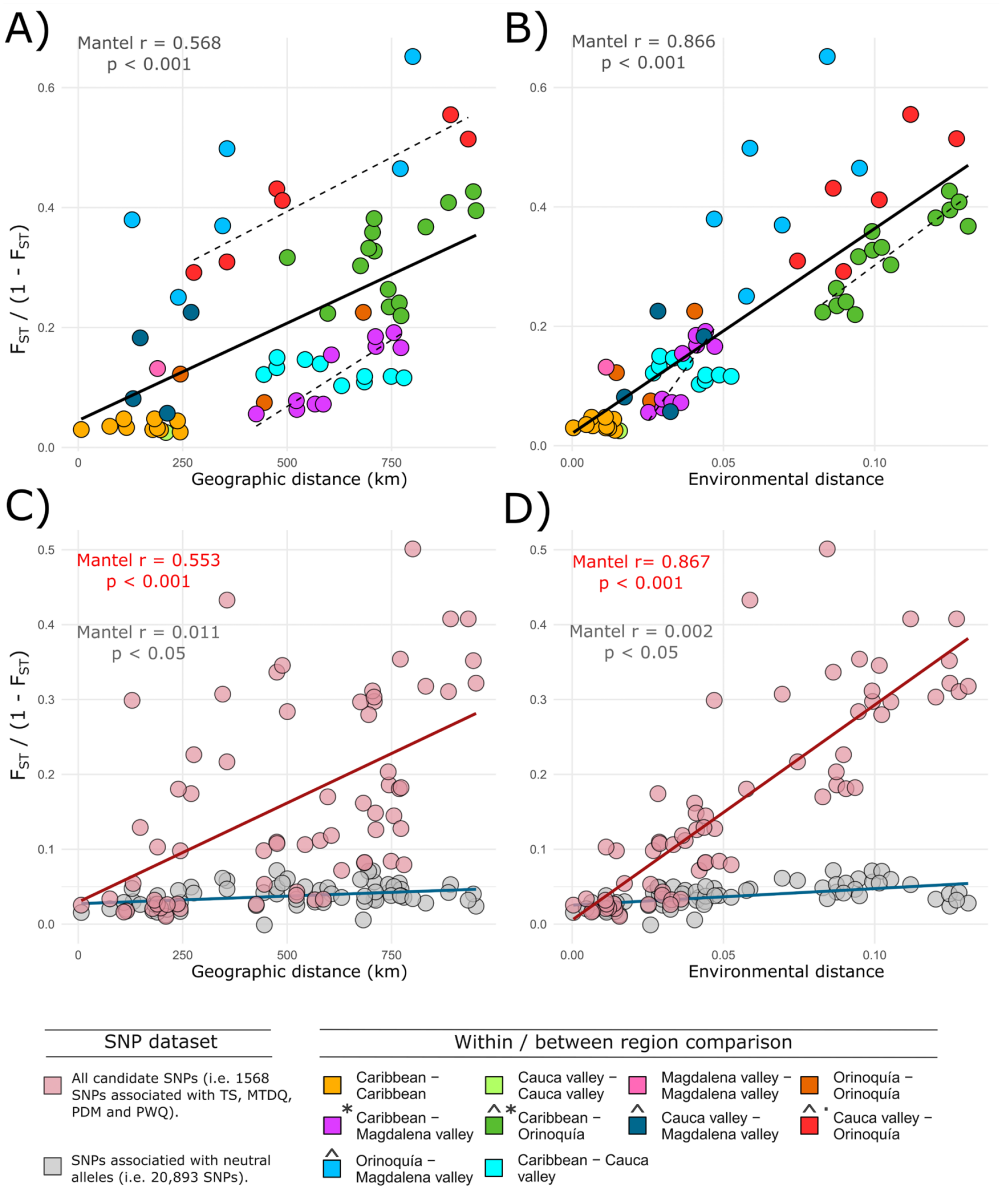
Test of genetic isolation by two distance methods, (A) Correlation between geographic distance and genetic distance for all SNPs (i.e., 29,266 SNPs). (B) Correlation between environmental distance and genetic distance for all SNPs. (C) Correlation between geographic distance and genetic distance for candidate SNPs (i.e., 1568 SNPs associated with TS, MTDQ, PDM and PWQ [pink]) versus SNPs associated with neutral alleles (i.e., 20,893 SNPs—gray). (D) Correlation between environmental distance and genetic distance for candidate SNPs (pink) versus SNPs associated with neutral alleles (gray). Colored dots in A and B indicate pairwise comparisons within‐ and between‐regions. Solid lines (i.e., black, red and blue lines) indicate regression fits shown for all significant correlations from Mantel tests. Dashed lines indicate regression fits for significant within‐ and between‐region comparisons. TS = temperature seasonality. MTDQ = average monthly mean temperature over the driest quarter of the year. PDM = precipitation during the driest month. PWQ = average monthly precipitation during the warmest quarter of the year. Asterisks (*) and (.) denote statistically significant and positive Mantel test correlations (*p* < 0.05 and *p* < 0.1) among inter‐regional comparisons. The “^” symbol indicates regional pairs geographically separated by the Andes.

A model of environmental isolation better explained genomic differentiation *F*_ST_/(1 − *F*_ST_) across the landscape (Figure 3B) than a model of only isolation by distance (Figure 3A), supporting H1. Furthermore, a stronger effect of geographic and environmental distances on genetic differentiation was found for candidate SNPs compared to SNPs associated with neutral alleles (Figure 3C,D). Our results indicate a significant (*p* < 0.05) role of resistance against gene flow for some regional‐pair comparisons (Caribbean‐Orinoquía, Caribbean‐Magdalena valley, Cauca valley‐Orinoquía) (Figure 3A,B), highlighting the Andes (i.e., mainly the Eastern cordillera) as a barrier to gene flow.

### 3.2 Local Adaptation to Drought and Heat Stress

Based on our conservative thresholds for candidate SNP detection, 5% (*n* = 1568) out of the total 29,266 SNPs were associated with temperature and precipitation variables (Figure S3) potentially resulting from local adaptation to climate and supporting H2. Specifically, 441 SNPs were associated with temperature seasonality (46 SNPs enriched for adaptive alleles associated with high seasonality and 395 SNPs enriched for adaptive alleles associated with low seasonality), 454 associated with precipitation of the driest month (413 SNPs enriched for adaptive alleles associated with wetter conditions, and 41 SNPs enriched for adaptive alleles associated with drier conditions), 93 associated with average monthly precipitation during the warmest quarter of the year (74 SNPs enriched for adaptive alleles associated with wetter conditions, and 19 SNPs enriched for adaptive alleles associated with drier conditions), and 180 associated with average monthly mean temperature over the driest quarter of the year (96 SNPs enriched for adaptive alleles associated with high temperatures, and 84 SNPs enriched for adaptive alleles associated with low temperatures).

**FIGURE 4:**
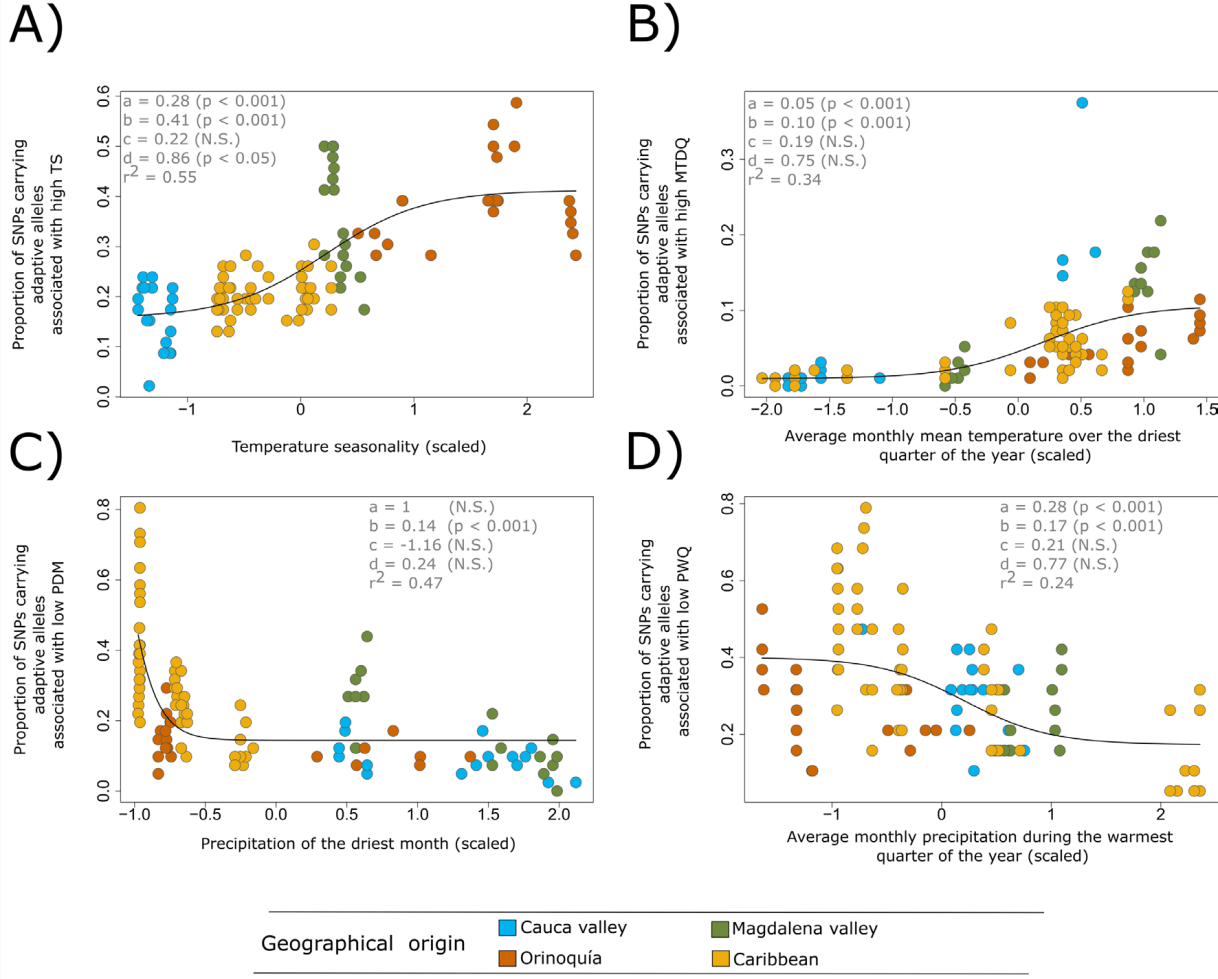
Variation in the per‐individual proportion of candidate SNPs carrying adaptive alleles across climatic gradients in *Enterolobium cyclocarpum* . Significant and stronger transitions were found for SNPs carrying adaptive alleles associated with high temperature seasonality. Circles represent individuals and are colored according to the region they were sampled. The *y*‐axis shows the proportion of candidate SNPs for which individuals carry at least one copy of the adaptive allele, and the *x*‐axis shows the scaled climatic variable (A–D). Black sigmoidal clines represent fitted trends across the environmental gradients. Parameter estimates (i.e., a, b, c and d) and *r*‐squared values are shown in gray at the top of each panel. TS = temperature seasonality. MTDQ = average monthly mean temperature over the driest quarter of the year. PDM = precipitation during the driest month. PWQ = average monthly precipitation during the warmest quarter of the year. Freq., frequency; N.S., non‐significant (i.e., *p* > 0.1); *p*, *p*‐value.

Furthermore, the proportion of per‐individual candidate SNPs carrying adaptive alleles, as well as the population allele frequencies at candidate SNPs, showed clinal variation and matched local climatic conditions corresponding to environments experiencing greater heat or drought stress (see Figure 4 and Figures S4–S11) indicating local adaptation to heat and drought conditions across the landscape, consistent with H2. Specifically, we show that individuals with high proportions of SNPs carrying adaptive alleles related to heat stress (i.e., correlated with increasing temperatures), and drought stress (i.e., correlated with decreasing precipitation) were found in localities with, respectively, the highest temperatures (i.e., heat stress, Figure 4A,B) and the lowest precipitation (i.e., drought stress, Figure 4C,D), supporting a local adaptation scenario. The strongest and best supported transition was observed for temperature seasonality, where the proportion of candidate SNPs carrying adaptive alleles changed significantly across the gradient with a well‐defined transition (upper limit [a] = 0.28, *t*‐value = 20.21, df = 165, *p*‐value < 0.001; lower limit [b] = 0.41, *t*‐value = 17.40, df = 165, *p*‐value < 0.001; cline width [d] = 0.86, *t*‐value = 2.63, df = 165, *p*‐value < 0.05). For the remaining climatic variables, clinal patterns showed weaker support, as non‐significant transitions were found for candidate SNPs carrying adaptive alleles associated with low average monthly precipitation during the warmest quarter of the year, low precipitation of the driest month and high monthly mean temperature over the driest quarter of the year (*p*‐values > 0.1 for most parameters), suggesting more gradual and less abrupt changes in the proportion of candidate SNPs carrying adaptive alleles across climatic gradients.

### 3.3 Genomic Offset and Gene Flow

**FIGURE 5:**
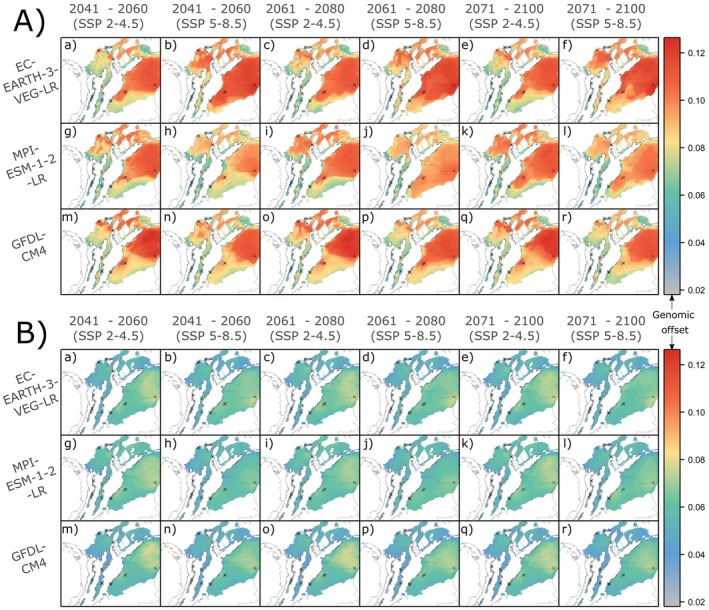
Genomic offset maps of *Enterolobium cyclocarpum* across main seasonally dry tropical forest regions in Northern South America (Colombia and parts of Venezuela) estimated from environmental—allele frequency associations. (A) Genomic offsets predicted using candidate SNPs enriched for adaptive alleles associated with increasing temperature (i.e., heat stress) and decreasing precipitation (i.e., drought stress) conditions (i.e., 202 SNPs). (B) Genomic offsets predicted using all candidate SNPs recovered (i.e., 1568 SNPs). Subpanels (a–r) show predictions across three different climate models (EC‐EARTH‐3‐VEG‐LR, MPI‐ESM‐1.2‐LR and GFDL‐CM4), two Shared Socioeconomic Pathway scenarios (SSP2‐4.5 and SSP5‐8.5) and three time periods (2041–2060, 2061–2080, and 2071–2100). Predicted genomic offsets were cropped to highlight main distribution of the species in the Caribbean, Cauca valley, Magdalena Valley and Orinoquía regions.

Inferences of genomic offset across the main areas of distribution (i.e., Caribbean, Magdalena valley, Cauca valley, and Orinoquía, as indicated in Figure 5) suggest varying degrees of genomic offset across the landscape, supporting H3. Genomic offsets were generally consistent across climate models, scenarios and time‐periods, with only minor spatial variation among climate models (Figure 5 and Figures S12 and S13). Specifically, when pooling results across the two candidate SNP datasets, as well as across all time periods, scenarios and climate models, regionally averaged genomic offsets across sampling localities were: 0.045 ± 0.015 (0.034 ± 0.006 for all candidate SNPs and 0.058 ± 0.010 for the set associated with only stressful conditions) in the Cauca valley, 0.056 ± 0.012 (0.046 ± 0.04 for all candidate SNPs and 0.065 ± 0.009 for the set associated with only stressful conditions) in the Magdalena valley, 0.064 ± 0.022 (0.044 ± 0.006 for all candidate SNPs and 0.084 ± 0.011 for the set associated with only stressful conditions) in the Caribbean, and 0.079 ± 0.021 (0.062 ± 0.007 for all candidate SNPs and 0.096 ± 0.017 for the set associated with only stressful conditions) in the Orinoquía. Additionally, genomic offsets were lower when calculated using the full set of candidate SNPs (Figure 5B) compared to the subset enriched for adaptive alleles associated with drought and heat stress (Figure 5A). However, only the dataset associated with heat and drought stress conditions yielded higher offsets than those derived from neutral alleles (Figure 5 and Figures S12 and S13). This indicates that including adaptive alleles associated with wetter and cooler conditions slightly reduces estimates of genomic mal‐adaptation under future climate scenarios, suggesting heterogenous responses across the landscape (i.e., not all regions are projected to experience increased heat and drought). Additionally, neutral alleles, although yielding intermediate offsets, or higher (i.e., when compared to the set including all candidate SNPs), produced spatial patterns similar to those observed using the datasets associated with adaptive alleles, and yielded slightly higher offsets when using the larger neutral subset (i.e., the set matching in size to the full set of candidate SNPs, Figure S13). In addition, genomic offsets were slightly higher under the SSP5‐8.5 scenario than under SSP2‐4.5 across all datasets, except when using all candidate SNPs (Figure S13). Our results show that across the entire current distribution in Colombia, *E. cyclocarpum* will face challenges to cope with new climatic conditions (i.e., genomic offsets greater than zero), and populations in Orinoquía are expected to be the most vulnerable, exhibiting the highest genomic offsets across all time periods (Figure 5 and Figure S13). These elevated offsets are widespread across the region and consistent across both SSP5‐8.5 and SSP2‐4.5 scenarios as well as all three climate models. Moreover, other areas captured in the models where the species also occurs (but unsampled, such as the dry and gallery forests in the Orinoquía in Venezuela) and non‐sampled localities in the Caribbean and the Magdalena valley, are also predicted to be at risk under most modeled scenarios (Figure 5A).

**FIGURE 6:**
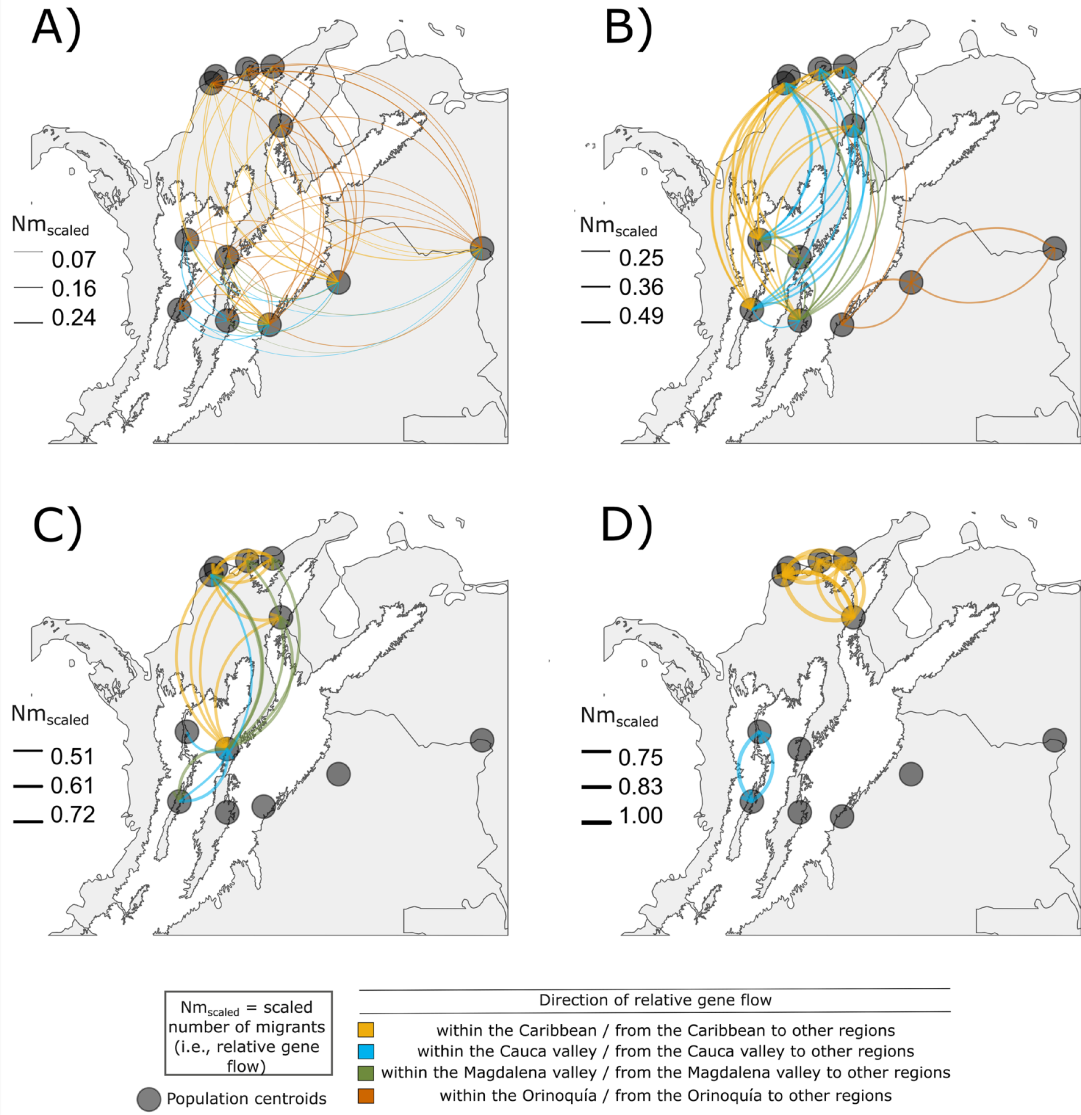
Relative gene flow (i.e., scaled number of migrants [*N*_*m* scaled_]) between populations of *Enterolobium cyclocarpum* in the Caribbean, inter‐Andean valleys and the Orinoquía in Colombia. (A) low relative gene flow (between 0 and 0.25), (B) low to intermediate relative gene flow (between 0.25 and 0.5), (C) intermediate to high relative gene flow (0.5–0.75), (D) high relative gene flow (0.75–1). Gray circles represent centroids of sampled sites for each of the 12 sampling localities across the four main regions (i.e., Caribbean, Cauca valley, Magdalena valley, and Orinoquía). Lowland areas are colored in gray and mountain regions (i.e., Andes) in white. Arrows and colors indicate the direction of gene flow as indicated in the legend at the bottom of the figure. Line thickness in the colored arrows represents the strength of relative gene flow.

In addition, high levels of relative gene flow were detected among sampling localities within the Caribbean and Cauca valley regions (e.g., mean of 0.8 ± 0.1 *N*_*m* scaled_ among localities in the Caribbean and 0.8 ± 0.03 *N*_*m* scaled_ among localities in the Cauca Valley; Figure 6D and Figure S14). In contrast, populations within the Magdalena valley and the Orinoquía regions exhibited low to intermediate levels of relative gene flow (e.g., mean of 0.4 ± 0.02 *N*_*m* scaled_ among localities of the Magdalena valley and 0.3 ± 0.1 *N*_*m* scaled_ among localities in the Orinoquía; Figure 6A,B and Figure S14). Relative gene flow between populations isolated by the Eastern Cordillera of the Andes was low (e.g., mean of 0.14 ± 0.04 *N*_*m* scaled_ from the Cauca valley to the Orinoquía and 0.15 ± 0.03 *N*_*m* scaled_ in the opposite direction; 0.14 ± 0.05 *N*_*m* scaled_ from the Magdalena valley to the Orinoquía and 0.15 ± 0.04 *N*_*m* scaled_ in the opposite direction; 0.17 ± 0.05 *N*_*m* scaled_ from the Caribbean to the Orinoquía, and 0.2 ± 0.04 *N*_*m* scaled_ in the opposite direction; Figure 6A and Figure S14), while relative gene flow across the Central Cordillera was low to intermediate (e.g., mean of 0.4 ± 0.2 *N*_*m* scaled_ both from the Cauca to the Magdalena valley and in the opposite direction), suggesting more limited dispersal across the Eastern cordillera than across the Central cordillera. Similarly, low to intermediate relative gene flow was observed across lowland humid forests adjacent to the Caribbean and inter‐Andean valleys (e.g., mean of 0.4 *N*_*m* scaled_ both from the Caribbean to the Cauca valley and in the opposite direction; mean of 0.5 *N*_*m* scaled_ from the Upper Magdalena Valley to the Caribbean, and 0.4 *N*_*m* scaled_ in the opposite direction; Figure 6A and Figure S14), suggesting genetic exchange across wet forests. In addition, intermediate to high relative gene flow was detected between the Middle Magdalena valley, Cauca valley and Caribbean populations (*N*_*m* scaled_ ranging from 0.55 to 0.72; Figure 6C); consistent with cases where individuals sampled in cattle ranches of the Middle Magdalena valley are genetically assigned to the Cauca Valley, or exhibit strong Caribbean ancestry (Figure 2A,B), suggesting cattle translocation across regions. Together, high genomic offsets combined with low relative gene flow indicate that sampling localities from the Orinoquía in particular will likely face challenges in coping with climate change in the future. Conversely, the remaining sites might be less vulnerable due to their lower genomic offsets and/or higher relative gene flow between populations.

## 4 Discussion

Our study provides evidence of genetic local adaptation in *E. cyclocarpum* across seasonally dry tropical forests of Colombia. We identified four genomic clusters, with differentiation shaped by isolation by distance and the Andean mountains. Drought and heat stress emerged as key drivers of adaptive variation, resulting in populations with genomic signatures of local climate adaptation. Genomic offsets were predicted to be highest in the Orinoquía region in eastern Colombia, where average gene flow within sampling localities was the lowest, suggesting that neither dispersal nor local adaptation may buffer these populations against future climate change. Despite these challenges, trees in the Orinoquía carry a high proportion of adaptive alleles linked to temperature seasonality and form a clearly differentiated genetic cluster, highlighting their value as a reservoir for future adaptation. These findings provide insights into how long‐lived tree species from threatened ecosystems such as Neotropical Seasonally Dry Forests (NSDFs) may respond to climate change, providing important baseline data to guide conservation efforts. Below, we discuss our main findings in the context of contemporary and historical population structure and admixture, local climate adaptation, and connectivity, and outline the conservation implications for NSDFs.

### 4.1 Contemporary and Historical Distribution and Admixture of *E. cyclocarpum*

The four genomic clusters we identified in *E. cyclocarpum* (Caribbean, Cauca valley, Upper Magdalena valley, and Orinoquía) reflect genetic differentiation among seasonally dry tropical forest enclaves separated by the eastern and central Andean cordilleras and intervening rain forests. These patterns are consistent with models of isolation by geographical and environmental distance (Figure 3) and with the geographically structured patterns of taxonomic and phylogenetic diversity across the disjunct distribution of NSDFs as a whole (DRYFLOR et al. 2016). In contrast to this pattern of strong geographical genetic differentiation, we detected evidence for substantial admixture of genotypes from populations in the Middle Magdalena valley. This high degree of admixture likely reflects the lesser degree of geographic, environmental and genetic isolation of this population cluster, or possible translocation of seed via livestock across regions (Aguirre‐Morales et al. 2020), given that the Middle Magdalena populations occur within an area of cattle ranching. Cattle ranches have expanded at the expense of forests, driving rapid transformation of Colombian lowland inter‐Andean dry and humid forests into pasture since the mid‐19th century (Van Ausdal 2009). Despite similar historical landscape transformation to cattle ranching in the Orinoquía (Etter 2015), trees from that region are genetically distinct and show low admixture coupled with local adaptation to high temperature seasonality regimes. This suggests natural occurrence and persistence in the Orinoquía (particularly in seasonally dry tropical forest relicts close to the piedmont), and reduced influx of non‐Orinoquía genotypes by cattle translocation across the country (or other dispersal agents). Moreover, as *E. cyclocarpum* is not fire‐adapted (Janzen 1988), its presence further east in the Orinoquía is apparently restricted to fire‐free habitats such as gallery forests and agricultural landscapes interspersed within the fire‐prone savanna, where the species might have expanded more recently following human transformation of savanna landscapes.

Given the recency of the uplift of the northern Andes in the late to mid‐Miocene to early Pliocene (ca. 10–4.5 Ma; Hoorn et al. 2010) and macrofossil evidence suggesting the existence of an older dry forest from the Esmeraldas formation in the Middle Magdalena valley of Colombia in the mid‐ to late‐Eocene (ca. 47.3–33.9 Ma; Martínez et al. 2021), it is likely that uplift of the northern Andes contributed to vicariant fragmentation of a once more continuous seasonally dry tropical forest, isolating populations across the lowlands and supporting a role of the Andes in shaping genetic differentiation of NSDFs populations. Furthermore, fossil wood identified as *Enterolobium* (Pérez‐Lara et al. 2021) from the Eocene El Bosque formation in Mexico suggests a long evolutionary history for the genus, consistent with more recent vicariant isolation of *E. cyclocarpum* populations associated with the northern Andean uplift. Similar patterns of genetic differentiation associated with geographical isolation across the Andes have been reported in other taxonomic groups, including invertebrates (Salgado‐Roa et al. 2018; Montejo‐Kovacevich et al. 2022), and vertebrates (Brumfield and Edwards 2007; Weir and Price 2011). In plants, studies of inter‐Andean seasonally dry tropical forests indicate that long‐term isolation in the central Andean dry valleys of southern Ecuador and Perú has promoted divergence through allopatric processes (Pennington et al. 2010; Särkinen et al. 2012). NSDFs could also have expanded during drier climates in the Last Glacial Maximum (LGM) (Pennington et al. 2000) and subsequently contracted. Such cycles of expansion and contraction may have played a key role in structuring regional populations (Burnham and Graham 1999; Mayle 2004), reinforcing genetic differentiation among population clusters during phases of habitat contraction.

### 4.2 Regional Patterns of Local Adaptation, Population Connectivity and Vulnerability

Our study provides evidence for local adaptation of populations of *E. cyclocarpum* across seasonally dry tropical forests of Colombia, shaped by both broad‐scale climatic gradients and finer‐scale environmental variation. Populations in the Caribbean and inter‐Andean valleys carry higher frequencies of adaptive alleles associated with temperature seasonality and low precipitation, indicating better adaptation to current heat and drought conditions. In contrast, populations in the Orinoquía show high frequencies of adaptive alleles linked to temperature fluctuations but fewer associated with prolonged heat and drought, suggesting limited adaptive potential under projected climate scenarios. These patterns highlight that local adaptation is not uniform across regions and may be constrained by fragmented distributions or climatically extreme landscapes. Evidence from other Neotropical trees supports these patterns, with species from both savannas and NSDFs in Brazil showing genetic adaptation to climatic stress, although savanna species appear more vulnerable to drought than NSDF species (Collevatti et al. 2019; Sobreiro et al. 2021; Vieira et al. 2022).

Genomic offset predictions reinforce this interpretation. Caribbean and inter‐Andean valley populations exhibited intermediate to low offsets under projected climates, suggesting greater capacity to cope with warming and drought, whereas Orinoquía populations displayed intermediate to high offsets, indicating increased vulnerability. Mal‐adaptation is predicted even in the near term (2041–2060), with similar challenges extending into mid‐ and long‐term projections (2061–2100), emphasizing the urgency of conservation interventions to maintain adaptive potential. Offsets were higher when calculated using candidate SNPs enriched for adaptive alleles associated with heat and drought stress than subsets associated with all candidate SNPs, and higher than offsets based on neutral alleles, although spatial patterns remained consistent. SNPs associated with neutral alleles showed intermediate but similar spatial trends, reflecting partial environmental structuring (Excoffier and Ray 2008; Forester et al. 2018).

Landscape‐scale habitat connectivity appears central to maintain adaptive potential. Well‐connected forest patches in the Caribbean region support higher gene flow, moderate to high frequencies of climate‐associated adaptive alleles, and low to intermediate genomic offsets, whereas the more fragmented Orinoquía populations exhibit reduced genetic connectivity and lower adaptive potential. Although gene flow can promote the spread of adaptive alleles, it may also introduce mal‐adaptive variants (Aguirre‐Liguori et al. 2021; Gougherty et al. 2021; Martin et al. 2023), particularly in landscapes influenced by livestock‐mediated seed dispersal. Furthermore, individuals from cattle pastures have been shown to exhibit reduced seed production and higher rates of fruit abortion compared to those growing in preserved forests (Rocha and Aguilar 2001), presumably attributable to impacts of disturbance on pollinators. Conservation strategies should therefore aim to maintain or restore connectivity among environmentally similar populations while minimizing risks of mal‐adaptive gene flow between populations with distinct environmental adaptations.

Finally, while candidate SNPs are strongly climate‐associated, some may reflect neutral demographic processes, but the impacts of spatial autocorrelation complicate disentangling climate‐driven from geographic variation (Excoffier and Ray 2008; Forester et al. 2018). Experimental validation and functional analyses, particularly for traits involved in drought and heat tolerance, are needed to confirm adaptive significance (Hancock et al. 2011; Fitzpatrick et al. 2021). Overall, these results indicate that preserving regionally distinct populations, maintaining habitat heterogeneity, and enhancing connectivity will be critical to safeguarding the adaptive capacity of *E. cyclocarpum* and other NSDF tree species under ongoing and future climate change across the highly threatened Neotropical Seasonally Dry Forest biome (Barrett and Schluter 2008; Christmas et al. 2016; DRYFLOR et al. 2016).

### 4.3 Limitations and Future Prospects

Our results should be interpreted with some caveats. First, our sampling covers only part of the species distribution, and the use of ddRAD sequencing provides a reduced representation of the genome; together, these factors clearly limit detection of the full spectrum of adaptive genetic variation within the species, which becomes even more relevant considering that natural adaptation is polygenic (Bomblies and Peichel 2022).

Second, unsampled populations could contribute beneficial alleles through gene flow, enhancing adaptive potential, or conversely constrain local adaptation via mal‐adaptive gene flow, whereby the influx of non‐locally adapted alleles may reduce fitness under changing environmental conditions (Kirkpatrick and Barton 1997; Fitzpatrick and Reid 2019; Lenormand 2002; Martin et al. 2023). In *E. cyclocarpum* , populations in fragmented landscapes or across steep climatic gradients may be particularly vulnerable, as gene flow from climatically divergent regions could swamp locally adaptive alleles and undermine responses to increasing heat and drought. Such dynamics may limit the ability of populations to track shifting environmental optima, even if connectivity is otherwise promoted to maintain genetic variation. On the other hand, considering the strong isolation by environment and isolation by distance observed for candidate SNPs, populations at nearby sites along environmental clines may already contain adaptive alleles for the near future (Höfner et al. 2025).

And third, our approach focuses on genotype‐environment associations but does not directly link adaptive alleles to functional traits or fitness, limiting our ability to infer the biological mechanisms underlying adaptation or the fitness consequences of the predicted genomic offsets. Although existing evidence from common garden experiments suggests a negative relationship between genomic offset and fitness (Capblancq et al. 2020; Fitzpatrick et al. 2021; Verrico et al. 2026), such tests remain scarce and have yet to be conducted for *E. cyclocarpum* .

Future research should integrate the risks of mal‐adaptive gene flow with genomic offset predictions to better forecast adaptive potential under climate change. This includes identifying source populations that carry pre‐adapted alleles without introducing mal‐adaptive variation, and considering how connectivity interventions (e.g., assisted gene flow or restoration) may differentially affect populations across regions such as the Caribbean versus the Orinoquía. Explicitly accounting for mal‐adaptive gene flow will help refine conservation strategies, ensuring that efforts to maintain or restore connectivity maximize adaptive benefits while minimizing potential genetic risks under accelerating environmental change (Aguirre‐Liguori et al. 2021).

In addition, alternative genomic offset metrics can capture mal‐adaptation under different contexts beyond the metrics considered here. For instance, forward genomic offset calculates the minimum predicted offset assuming potential dispersal to any location in space, whereas reverse offset predicts whether any population in the current range is pre‐adapted to a particular location under future climate conditions (Gougherty et al. 2021). Together, these complementary metrics may help identify source populations for assisted gene flow or restoration efforts, as well as regions where potential natural dispersal is unlikely to be sufficient to cope with future climate change. Furthermore, responses to climate change are often constrained and may not keep pace with the rate of environmental change due to limitations in genetic variation, demographic processes, phenotypic plasticity, dispersal, and pollination, which are crucial to predict more accurate responses under climate change (Nicotra et al. 2010; Aguirre‐Liguori et al. 2021; Chen et al. 2022; Martin et al. 2023). Taken together, these considerations indicate that integrating genomic offsets with complementary approaches such as common garden and reciprocal transplant experiments, genotype–phenotype analyses, and demographic studies can improve predictions of population responses and better inform restoration strategies under future climate change.

## 5 Conclusion

Our study revealed that *E. cyclocarpum* populations across Colombian seasonally dry tropical forests exhibit genomic signatures of local adaptation to heat and drought stress, shaped by climatic gradients, geographic barriers such as the Andes and historical environmental change. This adaptive variation facilitates resilience to climate change; however, populations in fragmented and poorly connected landscapes may lack the adaptive potential to cope with future increased heat and drought. Specifically, we highlight the risk of losing populations that contain irreplaceable adaptive alleles that may be beneficial under accelerating climate and land‐use change scenarios in the future. We advocate further research to improve our understanding of variation in Neotropical tree species using genomic approaches and highlight the need for conservation strategies that consider the adaptive potential of species, particularly in threatened biomes such as NSDFs. Finally, we emphasize the importance of preserving population connectivity across environmental gradients to maintain gene flow and facilitate adaptive responses (Christmas et al. 2016), as well as protecting a mosaic of regions that encompass the full range of a species' climatic and ecological variability (Barrett and Schluter 2008). Taken together, our findings contribute to predictive frameworks to understand responses to climate change in one of the world's most threatened biomes.

## Funding

This research was supported by the German Centre for Integrative Biodiversity Research (iDiv) Halle‐Jena‐Leipzig, funded by the German Research Foundation (DFG‐FZT 118, 202548816).

## Disclosure

Plant material was collected during the years 2020 and 2021in Colombia under collection permit 00589 granted by the Autoridad Nacional de Licencias Ambientales (National Authority of Environmental Licenses ‐ANLA): “Permiso Marco de Recolección de Especímenesde Especies Silvestres de la Diversidad Biológica con Fines de Investigación Científica No Comercial (Resolución 00589 del 10 de Abril de 2019.)”

## Conflicts of Interest

The authors declare no conflicts of interest.

## Supporting information


**File S1:** Correlations between candidate SNPs identified by partial redundancy analysis (RDA).

**Figure S1:** Pairwise correlations among environmental predictors used in the genotype‐environment association analyses: precipitation of the driest month in kg/m^2^ × month, average monthly precipitation during the warmest quarter of the year in kg/m^2^ × month, temperature seasonality (standard deviation of the monthly mean temperatures) in °C/100, average monthly mean temperature over the driest quarter of the year in °C. Histograms along the diagonal show the distribution of each variable across the 109 individuals sampled. Scatterplots in the lower triangle illustrate pairwise relationships, with the red lines indicating regression lines (red) with 95% confidence intervals (gray). The upper triangle reports Pearson correlation coefficients, quantifying the linear association between pairs of predictors.
**Figure S2:** Cross‐validation (CV) scores obtained from SNMF (Sparse Non‐negative Matrix Factorization) are depicted in the *y*‐axis for different numbers of inferred genetic clusters (K = 1–10) depicted in the *x*‐axis. CV scores decrease sharply from K = 1 to K = 4, and increase thereafter, suggesting that K = 4 best explains the population genetic structure.
**Figure S3:** Distribution of candidate SNPs based on the results from the partial redundancy analysis. Candidate SNPs (i.e., those showing genotype‐environment associations) correspond to those located at the tails of the distribution of the loadings on each canonical axis using a threshold of 2.8 standard deviations + − the mean [loading]. Candidate SNPs are depicted as colored dots across different canonical axis combinations: (A) axis 1 vs. 2, (B) axis 1 vs. 3, (C) axis 1 vs. 4, (D) axis 2 vs. 3, (E) axis 2 vs. 4, (F) axis 3 vs. 4, with axes 1 and 2 explaining most of the variation across candidate SNPs. Candidate SNPs are colored according to the climatic variable showing the strongest correlation with each SNP genotype: red colored SNPs correspond to those most strongly associated with average monthly mean temperature over the driest quarter of the year, green colored SNPs correspond to those most strongly associated to average monthly precipitation during the warmest quarter of the year, purple colored SNPs correspond to those most strongly associated with precipitation of the driest month, and yellow colored SNPs correspond to those most strongly associated with temperature seasonality. White dots represent non‐candidate SNPs. Black arrows depict the direction and strength of the correlation with the corresponding climatic variable labeled next to the arrow.
**Figure S4:** Per‐individual frequency of SNP loci carrying one or two putatively adaptive alleles associated with high or low temperature seasonality (TS). Circles represent individual samples. The y‐axis shows standardized TS values (purple) and per‐individual frequencies of SNP loci carrying adaptive alleles associated with high TS (yellow) or low TS (light green). Populations are arranged along the *x*‐axis and grouped by major geographic regions (Caribbean, Orinoquía, and inter‐Andean valleys). Climatic values and frequencies were standardized to a 0–1 range (i.e., minimum value is transformed to 0 and maximum to 1), enabling direct comparison of relative variation in TS and frequencies of candidate SNP loci carrying adaptive alleles associated with high or low TS across populations.
**Figure S5:** Per‐individual frequency of SNP loci carrying one or two adaptive alleles associated with high or low precipitation during the driest month (PDM). Circles represent individual samples. The y‐axis shows standardized PDM values (purple) and per‐individual frequencies of SNP loci carrying adaptive alleles associated with high PDM (light green) or low PDM (yellow). Populations are arranged along the *x*‐axis and grouped by major geographic regions (Caribbean, Orinoquía, and inter‐Andean valleys). Climatic values and frequencies were standardized to a 0–1 range (i.e., minimum value is transformed to 0 and maximum to 1), enabling direct comparison of relative variation in PDM and frequencies of SNP loci carrying adaptive alleles associated with high or low PDM across populations.
**Figure S6:** Per‐individual frequency of SNP loci carrying adaptive alleles associated with high and low average monthly precipitation during the warmest quarter of the year (PWQ). Circles represent individual samples. The *y*‐axis shows standardized PWQ values (purple) and per‐individual frequencies of SNP loci carrying adaptive alleles associated with high PWQ (light green) or low PWQ (yellow). Populations are arranged along the *x*‐axis and grouped by major geographic regions (Caribbean, Orinoquía, and inter‐Andean valleys). Climatic values and frequencies were standardized to a 0–1 range (i.e., minimum value is transformed to 0 and maximum to 1), enabling direct comparison of relative variation in PWQ and frequencies of SNP loci carrying adaptive alleles associated with high or low PWQ across populations.
**Figure S7:** Per‐individual frequency of SNP loci carrying one or two adaptive alleles associated with high and low average monthly mean temperature over the driest quarter of the year (MTDQ). Circles represent individual samples. The *y*‐axis shows standardized MTDQ values (purple) and per‐individual frequencies of SNP loci carrying adaptive alleles associated with high MTDQ (yellow) or low MTDQ (light green). Populations are arranged along the *x*‐axis and grouped by major geographic regions (Caribbean, Orinoquía, and inter‐Andean valleys). Climatic values and frequencies were standardized to a 0–1 range (i.e., minimum value is transformed to 0 and maximum to 1), enabling direct comparison of relative variation in MTDQ and frequencies of SNP loci carrying adaptive alleles associated with high or low MTDQ across populations.
**Figure S8:** Change in population allele frequencies for adaptive alleles associated with high TS (temperature seasonality) at candidate SNPs. We identified 46 candidate SNPs enriched for adaptive alleles associated with high TS. The dark blue line shows the tendency of frequency change based on a generalized additive model (GAM) across the gradient with 95% confidence intervals shown in light blue. Each population is represented by a black dot. Population allele frequencies are shown in the *y*‐axis. TS values are shown in the *x*‐axis. TS, temperature seasonality (standard deviation of the monthly mean temperatures) in °C/100.
**Figure S9:** Change in population allele frequencies for adaptive alleles associated with low PDM (precipitation of the driest month) at candidate SNPs. We identified 41 candidate SNPs enriched for putatively adaptive alleles associated with low PDM. The dark blue line shows the tendency of frequency change based on a generalized additive model (GAM) across the gradient with 95% confidence intervals shown in light blue. Each population is represented by a black dot. Population allele frequencies are shown in the *y*‐axis. PDM values are shown in the *x*‐axis. PDM = precipitation of the driest month in kg/m^2^ × month.
**Figure S10:** Change in population allele frequencies for adaptive alleles associated with high MTDQ (average monthly mean temperature over the driest quarter of the year) at candidate SNPs. We identified 96 candidate SNPs enriched for adaptive alleles associated with high MTDQ. The dark blue line shows the tendency of frequency change based on a generalized additive model (GAM) across the gradient with 95% confidence intervals shown in light blue. Each population is represented by a black dot. Population allele frequencies are shown in the *y*‐axis. MTDQ values are shown in the *x*‐axis. MTDQ = average monthly mean temperature over the driest quarter of the year in °C.
**Figure S11:** Change in population allele frequencies for adaptive alleles associated with low PWQ (average monthly precipitation during the warmest quarter of the year) at candidate SNPs. We identified 17 candidate SNPs enriched for adaptive alleles associated with low PWQ. The dark blue line shows the tendency of frequency change based on a generalized additive model (GAM) across the gradient with 95% confidence intervals shown in light blue. Each population is represented by a black dot. Population allele frequencies are shown in the *y*‐axis. PWQ values are shown in the *x*‐axis. PWQ = average monthly precipitation during the warmest quarter of the year in kg/m^2^ × month.
**Figure S12:** Genomic offset maps of *E. cyclocarpum* across its distribution in Northern South America (Colombia and parts of Venezuela) estimated from (A) environmental—allele frequency associations for SNPs associated with neutral alleles matching in size to the set associated with heat and drought stress conditions (i.e., 202 SNPs), and (B) environmental—allele frequency associations for SNPs associated with neutral alleles matching in size to the set of all candidate SNPs (i.e., 1568 SNPs). The association between allelic frequencies of the two neutral sets and current environment for the 12 populations studied was used to estimate genomic offsets using the “Gradient Forest” approach as described in the methods. Predicted genomic offsets were cropped to highlight main regions of the distribution of the species in the Caribbean, Cauca valley, Magdalena Valley and Orinoquía. Genomic offsets were calculated using three different climate models (EC‐EARTH‐3‐VEG‐LR, MPI‐ESM‐1.2‐LR and GFDL‐CM4), two Shared Socioeconomic Pathway scenarios (SSP2‐4.5 and SSP5‐8.8) and three time periods (2041–2060, 2061–2080, and 2071–2100). Individual samples are labeled with an “x”.
**Figure S13:** Genomic offsets across regions, under future climate projections for the periods 2041–2060, 2061–2080, and 2071–2100. Predictions are based on two Shared Socioeconomic Pathway scenarios (SSP2‐4.5 and SSP8‐8.5) and three climate models (EC‐EARTH‐3‐VEG‐LR, GFDL‐CM4, and MPI‐ESM‐1.2‐LR). Gray columns on the top represent the four geographical regions: Caribbean (first), Magdalena Valley (second), Cauca Valley (third), and Orinoquía (fourth). Gray panels on the right indicated the combination of time‐periods and emission scenarios. Genomic offsets were calculated using four data‐sets: (1) a set of SNPs associated with neutral alleles (blue) matching in number to the full candidate dataset (1568 SNPs). (2) a second set of SNPs also associated with neutral alleles (light blue) matching in number to the set of candidate SNPs enriched for adaptive alleles associated with heat and drought stress (202 SNPs). (3) All candidate SNPs identified in the partial RDA (i.e., 1568 SNPs associated with high or low PDM, PWQ, TS and MTDQ) (red). And (4) candidate SNPs enriched for adaptive alleles associated with heat or drought stress—202 SNP (pink). Dots represent twelve populations, ordered along the y‐axis according to their predicted genomic offset. Black horizontal lines within boxes indicate mean genomic offset per region. PDM = precipitation during the driest month. PWQ = average monthly precipitation during the warmest quarter of the year. TS = temperature seasonality. MTDQ = average monthly mean temperature over the driest quarter of the year.
**Figure S14:** Relative gene flow within regions and between pairs of regions. The regional pair involved is shown in the *x*‐axis. The arrows indicate the direction of gene flow. Relative gene flow is measured as the scaled number of migrants per generation (*N*_*m* scaled_) and is depicted in the *y*‐axis. Horizontal black lines within each box represent averages of relative gene flow. Dots represent pairwise comparisons between populations from two different regions or within the same region.
**Table S1:** Samples and corresponding climatic variables for individuals analyzed. Code = code of sampled individual in the field. Long. = decimal longitude. Lat. = decimal latitude. Region = geographic region of the sample. Locality = name of locality where the sample was taken. LOC. = Locality. PDM = precipitation of the driest month in kg/(m^2^ × month). PWQ = average monthly precipitation during the warmest quarter of the year in kg/(m^2^ × month). TS = temperature seasonality (standard deviation of the monthly mean temperatures) in °C/100. MTDQ = average monthly mean temperature over the driest quarter of the year in °C. Corresponding run accession IDs for the demultiplexed ddRAD sequences deposited in the European Nucleotide Archive (project accession number PRJEB112779) are provided in the first column.
**Table S2:** Description of the 12 sampling localities in Colombia.

## Data Availability

Demultiplexed ddRAD sequences were deposited in the European Nucleotide Archive (ENA) and are available under project accession number PRJEB112779. We provide the R script and input files required to reproduce all results in a DRYAD data and code repository (DOI: 10.5061/dryad.vhhmgqp8n).
