# Supplementary material for: Local Adaptation to Current but Not Future Climate in Seasonally Dry Tropical Forests: Population Genomic Evidence in a Neotropical Legume Tree
Source: Evol Appl. 2026 Aug 7;19(8):e70302. doi: 10.1111/eva.70302 (PMC13451525; doi:10.1111/eva.70302)
Supplement: Supplementary file 1 [file EVA-19-e70302-s002.docx]

**Supplementary Information for**

Local Adaptation to Current but Not Future Climate in Seasonally Dry Tropical Forests: Population Genomic Evidence in a Neotropical Legume Tree

**Francisco J. Velásquez-Puentes ^1,2,3*^, Walter Durka ^3,4^, Colin**

**Hughes ^5^, R. Toby Pennington ^6,7,8^, Jens J. Ringelberg ^9^, Renske E. Onstein ^7 3,10,11^, Christopher D. Barratt ^2,3,10,12^**

1. Universidad del Norte, Barranquilla, Colombia.

2. Leipzig University, Leipzig, Germany.

3. German Centre for Integrative Biodiversity Research (iDiv) Halle-Jena-Leipzig,

Leipzig, Germany.

4. Department of Community Ecology (BZF), Helmholtz Centre for Environmental

Research - UFZ, Halle, Germany.

5. Department of Systematic & Evolutionary Botany, University of Zurich, Zurich

Switzerland.

6. Department of Biology, Washington University, St Louis, USA.

7. Department of Geography, University of Exeter, Exeter, UK.

8. Missouri Botanical Garden, St Louis, USA.

9. Biosystematics Group, Wageningen University and Research,

Wageningen, the Netherlands.

10. Naturalis Biodiversity Center, Leiden, The Netherlands.

11. Institute of Biology Leiden (IBL), Leiden University,

Leiden, The Netherlands.

12. Animal Breeding and Genomics, Wageningen University and Research,

Wageningen, the Netherlands.

**Corresponding author:** Francisco J. Velásquez-Puentes.

e-mail: velasquez.puentes.francisco@gmail.com

**Shared senior authorship**: Renske E. Onstein and Christopher D. Barratt.

**Identification of SNPs Associated with Neutral Alleles**

SNPs associated with neutral alleles were obtained by excluding outlier SNPs exhibiting signatures of selection – those characterized by having large allelic frequencies between populations due to selection – from the set of original 29,266 SNPs. A principal component analysis was run on the whole set of SNPs (i.e. 29,266 SNPs) using the pcadapt function from the R package pcadapt (Luu et al. 2017, Privé et al. 2020). The pcadapt function uses z-scores from regressing SNP genotypes with principal components describing population structure. Then, it employs the squared Mahalanobis distance scaled by a genomic inflation factor to measure the distance of each SNP from the mean in multi-dimensional space (Luu et al. 2017). These scaled distances are used to identify outliers by assessing whether the vector of z-scores for a given SNP deviates significantly from the z-scores distribution, assigning p-values for each SNP (Luu et al. 2017). We executed the pcadapt function on the set of 29,266 SNPs, retaining the first four principal components. These components were selected as they explain the highest proportion of variance among populations in the data as inferred from the bend point on a curve of variance explained vs. number of principal components. A total of 25 SNPs were excluded by pcadapt prior to outlier detection because they were monomorphic among individuals with non-missing genotypes, retaining 29,241 polymorphic SNPs. Subsequently, p-values obtained for each SNP were transformed into q-values using the qvalue function from the R package qval (Storey & Tibshirani 2023), as q-values provide a more reliable measure of significance than p-values by quantifying the proportion of false positives incurred when a particular test is considered significant (i.e. false discovery rate). Outliers were classified as those SNP having q-values less than 0.1 (i.e. 8348 SNPs) and were removed from the set of 29,241 SNPs to obtain SNPs associated with neutral alleles (i.e. 20,893 SNPs).

**SNP Datasets and Corresponding Analyses**

SNP datasets associated with neutral alleles (i.e. 20,893 SNPs), candidate SNPs (i.e. 1568 SNPs identified by the partial RDA and outlier analysis), and the complete SNP dataset (i.e. 29,266 SNPs) were used to estimate pairwise genetic differentiation *F*_ST_ (Weir & Cockerham 1984). Pairwise genetic differentiation was subsequently transformed as *F*_ST_ / (1- *F*_ST_) and compared with geographic and environmental distances using Mantel tests, as described in the methods.

Genomic offsets were estimated with four SNP subsets. Subsets 1 and 2 were composed of SNPs associated with neutral alleles, whereas subsets 3 and 4 were composed of candidate SNPs (i.e., those showing genotype-environment associations). To facilitate comparisons, neutral subsets were matched in size to the adaptive subsets. Specifically, subsets 1 and 3 each contained 1568 SNPs; subset 3 comprised the complete set of candidate SNPs (i.e. those associated with and low: PDM, PWQ, TS and MTDQ), whereas subset 1 contained SNPs associated with neutral alleles. Likewise, subsets 2 and 4 each contained 202 SNPs; subset 4 comprised SNPs enriched for adaptive alleles associated with drought and heat stress, whereas subset 2 contained SNPs associated with neutral alleles. Candidate SNPs were derived from the partial RDA and threshold applied as described in the methods. Subsets 1 and 2 were randomly sampled from the larger dataset of 20,893 SNPs associated with neutral alleles obtained as described in the previous section.

**Supplementary Figures**

**Figure S1.** Pairwise correlations among environmental predictors used in the genotype-environment association analyses: precipitation of the driest month in Kg/m^2^*month, average monthly precipitation during the warmest quarter of the year in Kg/m^2^*month, temperature seasonality (standard deviation of the monthly mean temperatures) in ºC/100, average monthly mean temperature over the driest quarter of the year in ºC. Histograms along the diagonal show the distribution of each variable across the 109 individuals sampled. Scatterplots in the lower triangle illustrate pairwise relationships, with the red lines indicating regression lines (red) with 95% confidence intervals (gray). The upper triangle reports Pearson correlation coefficients, quantifying the linear association between pairs of predictors.

**Figure S2.** Cross-validation (CV) scores obtained from SNMF (Sparse Non-negative Matrix Factorization) are depicted in the y-axis for different numbers of inferred genetic clusters (K = 1 - 10) depicted in the x-axis. CV scores decrease sharply from K =1 to K = 4, and increase thereafter, suggesting that K = 4 best explains the population genetic structure.

**Figure S3.** Distribution of candidate SNPs based on the results from the partial redundancy analysis. Candidate SNPs (i.e. those showing genotype-environment associations) correspond to those located at the tails of the distribution of the loadings on each canonical axis using a threshold of 2.8 standard deviations +- the mean [loading]. Candidate SNPs are depicted as coloured dots across different canonical axis combinations: A) axis 1 vs. 2, B) axis 1 vs. 3, C) axis 1 vs. 4, D) axis 2 vs. 3, E) axis 2 vs. 4 , F) axis 3 vs. 4, with axes 1 and 2 explaining most of the variation across candidate SNPs. Candidate SNPs are coloured according to the climatic variable showing the strongest correlation with each SNP genotype: red coloured SNPs correspond to those most strongly associated with average monthly mean temperature over the driest quarter of the year, green coloured SNPs correspond to those most strongly associated to average monthly precipitation during the warmest quarter of the year, purple coloured SNPs correspond to those most strongly associated with precipitation of the driest month, and yellow coloured SNPs correspond to those most strongly associated with temperature seasonality. White dots represent non-candidate SNPs. Black arrows depict the direction and strength of the correlation with the corresponding climatic variable labeled next to the arrow.

**Figure S4.** Per-individual frequency of SNP loci carrying one or two putatively adaptive alleles associated with high or low temperature seasonality (TS). Circles represent individual samples. The y-axis shows standardized TS values (purple) and per-individual frequencies of SNP loci carrying adaptive alleles associated with high TS (yellow) or low TS (light green). Populations are arranged along the x-axis and grouped by major geographic regions (Caribbean, Orinoquía, and inter-Andean valleys). Climatic values and frequencies were standardized to a 0 – 1 range (i.e. minimum value is transformed to 0 and maximum to 1), enabling direct comparison of relative variation in TS and frequencies of candidate SNP loci carrying adaptive alleles associated with high or low TS across populations.

**Figure S5.** Per-individual frequency of SNP loci carrying one or two adaptive alleles associated with high or low precipitation during the driest month (PDM). Circles represent individual samples. The y-axis shows standardized PDM values (purple) and per-individual frequencies of SNP loci carrying adaptive alleles associated with high PDM (light green) or low PDM (yellow). Populations are arranged along the x-axis and grouped by major geographic regions (Caribbean, Orinoquía, and inter-Andean valleys). Climatic values and frequencies were standardized to a 0 – 1 range (i.e. minimum value is transformed to 0 and maximum to 1), enabling direct comparison of relative variation in PDM and frequencies of SNP loci carrying adaptive alleles associated with high or low PDM across populations.

**Figure S6.** Per-individual frequency of SNP loci carrying adaptive alleles associated with high and low average monthly precipitation during the warmest quarter of the year (PWQ). Circles represent individual samples. The y-axis shows standardized PWQ values (purple) and per-individual frequencies of SNP loci carrying adaptive alleles associated with high PWQ (light green) or low PWQ (yellow). Populations are arranged along the x-axis and grouped by major geographic regions (Caribbean, Orinoquía, and inter-Andean valleys). Climatic values and frequencies were standardized to a 0 – 1 range (i.e. minimum value is transformed to 0 and maximum to 1), enabling direct comparison of relative variation in PWQ and frequencies of SNP loci carrying adaptive alleles associated with high or low PWQ across populations.

**Figure S7**. Per-individual frequency of SNP loci carrying one or two adaptive alleles associated with high and low average monthly mean temperature over the driest quarter of the year (MTDQ). Circles represent individual samples. The y-axis shows standardized MTDQ values (purple) and per-individual frequencies of SNP loci carrying adaptive alleles associated with high MTDQ (yellow) or low MTDQ (light green). Populations are arranged along the x-axis and grouped by major geographic regions (Caribbean, Orinoquía, and inter-Andean valleys). Climatic values and frequencies were standardized to a 0 – 1 range (i.e. minimum value is transformed to 0 and maximum to 1), enabling direct comparison of relative variation in MTDQ and frequencies of SNP loci carrying adaptive alleles associated with high or low MTDQ across populations.

**Figure S8.** Change in population allele frequencies for adaptive alleles associated with high TS (temperature seasonality) at candidate SNPs. We identified 46 candidate SNPs enriched for adaptive alleles associated with high TS. The dark blue line shows the tendency of frequency change based on a generalized additive model (GAM) across the gradient with 95% confidence intervals shown in light blue. Each population is represented by a black dot. Population allele frequencies are shown in the y-axis. TS values are shown in the x-axis.. TS = temperature seasonality (standard deviation of the monthly mean temperatures) in ºC/100.

**Figure S9**. Change in population allele frequencies for adaptive alleles associated with low PDM (precipitation of the driest month) at candidate SNPs. We identified 41 candidate SNPs enriched for putatively adaptive alleles associated with low PDM. The dark blue line shows the tendency of frequency change based on a generalized additive model (GAM) across the gradient with 95% confidence intervals shown in light blue. Each population is represented by a black dot. Population allele frequencies are shown in the y-axis. PDM values are shown in the x-axis. PDM = precipitation of the driest month in Kg/m^2^*month.

**Figure S10**. Change in population allele frequencies for adaptive alleles associated with high MTDQ (average monthly mean temperature over the driest quarter of the year) at candidate SNPs. We identified 96 candidate SNPs enriched for adaptive alleles associated with high MTDQ. The dark blue line shows the tendency of frequency change based on a generalized additive model (GAM) across the gradient with 95% confidence intervals shown in light blue. Each population is represented by a black dot. Population allele frequencies are shown in the y-axis. MTDQ values are shown in the x-axis. MTDQ = average monthly mean temperature over the driest quarter of the year in ^º^C.

**Figure S11**. Change in population allele frequencies for adaptive alleles associated with low PWQ (average monthly precipitation during the warmest quarter of the year) at candidate SNPs. We identified 17 candidate SNPs enriched for adaptive alleles associated with low PWQ. The dark blue line shows the tendency of frequency change based on a generalized additive model (GAM) across the gradient with 95% confidence intervals shown in light blue. Each population is represented by a black dot. Population allele frequencies are shown in the y-axis. PWQ values are shown in the x-axis. PWQ = average monthly precipitation during the warmest quarter of the year in Kg/m^2^*month.

**Figure S12**. Genomic offset maps of *E. cyclocarpum* across its distribution in Northern South America (Colombia and parts of Venezuela) estimated from A) environmental - allele frequency associations for SNPs associated with neutral alleles matching in size to the set associated with heat and drought stress conditions (i.e. 202 SNPs), and B) environmental - allele frequency associations for SNPs associated with neutral alleles matching in size to the set of all candidate SNPs (i.e. 1568 SNPs). The association between allelic frequencies of the two neutral sets and current environment for the twelve populations studied was used to estimate genomic offsets using the “Gradient Forest” approach as described in the methods. Predicted genomic offsets were cropped to highlight main regions of the distribution of the species in the Caribbean, Cauca valley, Magdalena Valley and Orinoquía. Genomic offsets were calculated using three different climate models (EC-EARTH-3-VEG-LR, MPI-ESM-1.2-LR and GFDL-CM4), two Shared Socioeconomic Pathway scenarios (SSP2-4.5 and SSP5-8.8) and three time periods (2041-2060, 2061-2080, and 2071-2100). Individual samples are labelled with an “x”.

**Figure S13**. Genomic offsets across regions, under future climate projections for the periods 2041-2060, 2061-2080, and 2071-2100. Predictions are based on two Shared Socioeconomic Pathway scenarios (SSP2-4.5 and SSP8-8.5) and three climate models (EC-EARTH-3-VEG-LR, GFDL-CM4, and MPI-ESM-1.2-LR). Gray columns on the top represent the four geographical regions: Caribbean (first), Magdalena Valley (second), Cauca Valley (third), and Orinoquía (fourth). Gray panels on the right indicated the combination of time-periods and emission scenarios. Genomic offsets were calculated using four data-sets: 1) a set of SNPs associated with neutral alleles (blue) matching in number to the full candidate dataset (1568 SNPs). 2) a second set of SNPs also associated with neutral alleles (light blue) matching in number to the set of candidate SNPs enriched for adaptive alleles associated with heat and drought stress (202 SNPs). 3) all candidate SNPs identified in the partial RDA (i.e. 1568 SNPs associated with high or low PDM, PWQ, TS and MTDQ) (red). And 4) candidate SNPs enriched for adaptive alleles associated with heat or drought stress - 202 SNP (pink). Dots represent twelve populations, ordered along the y-axis according to their predicted genomic offset. Black horizontal lines within boxes indicate mean genomic offset per region. PDM = precipitation during the driest month. PWQ = average monthly precipitation during the warmest quarter of the year. TS = temperature seasonality. MTDQ = average monthly mean temperature over the driest quarter of the year.

**Figure S14.** Relative gene flow within regions and between pairs of regions. The regional pair involved is shown in the x-axis. The arrows indicate the direction of gene flow. Relative gene flow is measured as the scaled number of migrants per generation (N_m scaled_) and is depicted in the y-axis. Horizontal black lines within each box represent averages of relative gene flow. Dots represent pairwise comparisons between populations from two different regions or within the same region.

**Supplementary Tables**

**Table S1.** Samples and corresponding climatic variables for individuals analyzed. Code = code of sampled individual in the field. Long. = decimal longitude. Lat. = decimal latitude. Region = geographic region of the sample. Locality = name of locality where the sample was taken. LOC. = Locality. PDM = precipitation of the driest month in Kg/(m^2^*month). PWQ = average monthly precipitation during the warmest quarter of the year in Kg/(m^2^*month). TS = temperature seasonality (standard deviation of the monthly mean temperatures) in ºC/100. MTDQ = average monthly mean temperature over the driest quarter of the year in ºC. Corresponding run accession IDs for the demultiplexed ddRAD sequences deposited in the European Nucleotide Archive (project accession number **PRJEB112779**) are provided in the first column.

| **Run accession ID (ENA)** | **Sample** | **Long.** | **Lat** | **Region** | **Locality** | **LOC.** | **PDM** | **PWQ** | **TS** | **TDQ** |
| --- | --- | --- | --- | --- | --- | --- | --- | --- | --- | --- |
| ERR17367813 | FVP001 | -75.099069 | 10.759251 | Caribbean | Atlático - Piojó | Loc. 1 | 0.5 | 32.02 | 3.64 | 29.95 |
| ERR17367818 | FVP 002 | -75.09695 | 10.760322 | Caribbean | Atlático - Piojó | Loc. 1 | 0.5 | 32.02 | 3.64 | 29.95 |
| ERR17367821 | FVP 003 | -75.10024 | 10.753592 | Caribbean | Atlático - Piojó | Loc. 1 | 0.51 | 32.26 | 3.55 | 29.93 |
| ERR17367828 | FVP 004 | -75.099281 | 10.753314 | Caribbean | Atlático - Piojó | Loc. 1 | 0.52 | 32.6 | 3.48 | 29.95 |
| ERR17367833 | FVP 005 | -75.094473 | 10.756324 | Caribbean | Atlático - Piojó | Loc. 1 | 0.52 | 32.6 | 3.48 | 29.95 |
| ERR17368145 | FVP 006 | -75.097031 | 10.753963 | Caribbean | Atlático - Piojó | Loc. 1 | 0.52 | 32.6 | 3.48 | 29.95 |
| ERR17367846 | FVP 007 | -75.098992 | 10.753864 | Caribbean | Atlático - Piojó | Loc. 1 | 0.52 | 32.6 | 3.48 | 29.95 |
| ERR17367848 | FVP 008 | -75.102458 | 10.753413 | Caribbean | Atlático - Piojó | Loc. 1 | 0.51 | 32.26 | 3.55 | 29.93 |
| ERR17367851 | FVP 009 | -75.100695 | 10.76075 | Caribbean | Atlático - Piojó | Loc. 1 | 0.49 | 31.72 | 3.52 | 29.93 |
| ERR17367856 | FVP 010 | -75.0927 | 10.757319 | Caribbean | Atlático - Piojó | Loc. 1 | 0.52 | 32.6 | 3.48 | 29.95 |
| ERR17367858 | FVP 011 | -75.091744 | 10.758028 | Caribbean | Atlático - Piojó | Loc. 1 | 0.53 | 32.78 | 3.79 | 29.96 |
| ERR17367860 | FVP 012 | -75.090519 | 10.758112 | Caribbean | Atlático - Piojó | Loc. 1 | 0.53 | 32.78 | 3.79 | 29.96 |
| ERR17367883 | FVP 013 | -75.030931 | 10.751975 | Caribbean | Atlántico -Usiacurí | Loc. 2 | 0.53 | 20.81 | 4.01 | 29.98 |
| ERR17367885 | FVP 014 | -75.031569 | 10.752181 | Caribbean | Atlántico -Usiacurí | Loc. 2 | 0.53 | 20.81 | 4.01 | 29.98 |
| ERR17368134 | FVP 015 | -75.031978 | 10.752324 | Caribbean | Atlántico -Usiacurí | Loc. 2 | 0.53 | 20.81 | 4.01 | 29.98 |
| ERR17367904 | FVP 016 | -75.036576 | 10.75284 | Caribbean | Atlántico -Usiacurí | Loc. 2 | 0.53 | 20.92 | 3.64 | 29.97 |
| ERR17367908 | FVP 017 | -75.038556 | 10.752358 | Caribbean | Atlántico -Usiacurí | Loc. 2 | 0.53 | 20.92 | 3.64 | 29.97 |
| ERR17367910 | FVP 018 | -75.035423 | 10.753394 | Caribbean | Atlántico -Usiacurí | Loc. 2 | 0.53 | 20.92 | 3.64 | 29.97 |
| ERR17367915 | FVP 019 | -75.034344 | 10.753199 | Caribbean | Atlántico -Usiacurí | Loc. 2 | 0.53 | 20.92 | 3.64 | 29.97 |
| ERR17367925 | FVP 020 | -75.024604 | 10.749287 | Caribbean | Atlántico -Usiacurí | Loc. 2 | 0.53 | 20.68 | 3.83 | 30.01 |
| ERR17367931 | FVP 021 | -75.024516 | 10.74925 | Caribbean | Atlántico -Usiacurí | Loc. 2 | 0.53 | 20.68 | 3.83 | 30.01 |
| ERR17367934 | FVP 022 | -75.023816 | 10.747753 | Caribbean | Atlántico -Usiacurí | Loc. 2 | 0.53 | 20.68 | 3.83 | 30.01 |
| ERR17367936 | FVP 023 | -74.107951 | 11.146103 | Caribbean | Magdalena - Minca | Loc. 3 | 1.54 | 84.85 | 4.36 | 29.49 |
| ERR17368146 | FVP 024 | -74.109087 | 11.146023 | Caribbean | Magdalena - Minca | Loc. 3 | 1.48 | 83.35 | 4.54 | 29.57 |
| ERR17367944 | FVP 025 | -74.118228 | 11.132427 | Caribbean | Magdalena - Minca | Loc. 3 | 1.5 | 87.54 | 4.23 | 29.54 |
| ERR17367946 | FVP 026 | -74.119088 | 11.129088 | Caribbean | Magdalena - Minca | Loc. 3 | 1.5 | 87.54 | 4.23 | 29.54 |
| ERR17367949 | FVP 027 | -74.118423 | 11.1321 | Caribbean | Magdalena - Minca | Loc. 3 | 1.5 | 87.54 | 4.23 | 29.54 |
| ERR17367953 | FVP 029 | -74.104691 | 11.138808 | Caribbean | Magdalena - Minca | Loc. 3 | 1.56 | 86.49 | 4.36 | 29.51 |
| ERR17367955 | FVP 030 | -74.104793 | 11.138344 | Caribbean | Magdalena - Minca | Loc. 3 | 1.56 | 86.49 | 4.36 | 29.51 |
| ERR17367961 | FVP 031 | -74.119532 | 11.144762 | Caribbean | Magdalena - Minca | Loc. 3 | 1.43 | 82.05 | 4.48 | 29.62 |
| ERR17367964 | FVP 032 | -74.119551 | 11.144534 | Caribbean | Magdalena - Minca | Loc. 3 | 1.43 | 82.05 | 4.48 | 29.62 |
| ERR17367966 | FVP 034 | -73.431606 | 11.20302 | Caribbean | La Guajira - Mingueo | Loc. 4 | 2.78 | 49.71 | 5.83 | 29.94 |
| ERR17367968 | FVP 035 | -73.433475 | 11.20448 | Caribbean | La Guajira - Mingueo | Loc. 4 | 2.84 | 50.38 | 6.01 | 29.93 |
| ERR17367975 | FVP 036 | -73.433601 | 11.204346 | Caribbean | La Guajira - Mingueo | Loc. 4 | 2.84 | 50.38 | 6.01 | 29.93 |
| ERR17367977 | FVP 038 | -73.424381 | 11.2033 | Caribbean | La Guajira - Mingueo | Loc. 4 | 2.72 | 49.14 | 5.69 | 29.94 |
| ERR17368222 | FVP 039 | -73.423572 | 11.203961 | Caribbean | La Guajira - Mingueo | Loc. 4 | 2.72 | 49.14 | 5.69 | 29.94 |
| ERR17367990 | FVP 040 | -73.423682 | 11.204251 | Caribbean | La Guajira - Mingueo | Loc. 4 | 2.72 | 49.14 | 5.69 | 29.94 |
| ERR17367995 | FVP 041 | -73.419461 | 11.204279 | Caribbean | La Guajira - Mingueo | Loc. 4 | 2.72 | 49.14 | 5.69 | 29.94 |
| ERR17367998 | FVP 042 | -73.438675 | 11.191261 | Caribbean | La Guajira - Mingueo | Loc. 4 | 3 | 54.41 | 5.92 | 29.87 |
| ERR17368002 | FVP 047 | -73.407305 | 11.205214 | Caribbean | La Guajira - Mingueo | Loc. 4 | 2.6 | 47.73 | 5.85 | 29.96 |
| ERR17368005 | FVP 048 | -73.408172 | 11.205542 | Caribbean | La Guajira - Mingueo | Loc. 4 | 2.6 | 47.73 | 5.85 | 29.96 |
| ERR17367790 | FVP 050 | -73.166364 | 9.60118 | Caribbean | Cesar- La Jagua de Ibirico | Loc. 5 | 1.29 | 24.4 | 4.82 | 29.77 |
| ERR17367791 | FVP 051 | -73.16557 | 9.60033 | Caribbean | Cesar- La Jagua de Ibirico | Loc. 5 | 1.29 | 24.4 | 4.82 | 29.77 |
| ERR17367793 | FVP 052 | -73.164742 | 9.602538 | Caribbean | Cesar- La Jagua de Ibirico | Loc. 5 | 1.29 | 24.4 | 4.82 | 29.77 |
| ERR17367795 | FVP 053 | -73.196685 | 9.623482 | Caribbean | Cesar- La Jagua de Ibirico | Loc. 5 | 1.34 | 25.98 | 5.79 | 29.96 |
| ERR17367797 | FVP 054 | -73.230895 | 9.599737 | Caribbean | Cesar- La Jagua de Ibirico | Loc. 5 | 1.43 | 27.14 | 6.46 | 30.05 |
| ERR17367799 | FVP 056 | -73.228146 | 9.595369 | Caribbean | Cesar- La Jagua de Ibirico | Loc. 5 | 1.43 | 27.14 | 6.46 | 30.05 |
| ERR17367800 | FVP 057 | -73.228607 | 9.596004 | Caribbean | Cesar- La Jagua de Ibirico | Loc. 5 | 1.43 | 27.14 | 6.46 | 30.05 |
| ERR17367804 | FVP 058 | -73.193389 | 9.622444 | Caribbean | Cesar- La Jagua de Ibirico | Loc. 5 | 1.34 | 25.98 | 5.79 | 29.96 |
| ERR17367807 | FVP 059 | -73.183189 | 9.61667 | Caribbean | Cesar- La Jagua de Ibirico | Loc. 5 | 1.31 | 25.4 | 5.3 | 29.87 |
| ERR17367809 | FVP 060 | -73.190645 | 9.621543 | Caribbean | Cesar- La Jagua de Ibirico | Loc. 5 | 1.32 | 25.63 | 5.61 | 29.95 |
| ERR17368044 | FVP 061 | -74.661447 | 4.341206 | Upper Magdalena valley | Cundinamarca -Nilo | Loc. 6 | 5.23 | 51.49 | 6.5 | 29.79 |
| ERR17368045 | FVP 062 | -74.658074 | 4.336563 | Upper Magdalena valley | Cundinamarca -Nilo | Loc. 6 | 5.07 | 52.4 | 6.53 | 29.77 |
| ERR17368047 | FVP 063 | -74.655977 | 4.336669 | Upper Magdalena valley | Cundinamarca -Nilo | Loc. 6 | 5.07 | 52.4 | 6.53 | 29.77 |
| ERR17368049 | FVP 064 | -74.659484 | 4.334364 | Upper Magdalena valley | Cundinamarca -Nilo | Loc. 6 | 5.23 | 51.49 | 6.5 | 29.79 |
| ERR17368050 | FVP 065 | -74.66443 | 4.312066 | Upper Magdalena valley | Cundinamarca -Nilo | Loc. 6 | 5.41 | 52.64 | 6.28 | 29.8 |
| ERR17368051 | FVP 066 | -74.66561 | 4.3222 | Upper Magdalena valley | Cundinamarca -Nilo | Loc. 6 | 5.35 | 52.47 | 6.28 | 29.79 |
| ERR17368053 | FVP 067 | -74.661872 | 4.309509 | Upper Magdalena valley | Cundinamarca -Nilo | Loc. 6 | 5.41 | 52.64 | 6.28 | 29.8 |
| ERR17368054 | FVP 068 | -74.667007 | 4.331321 | Upper Magdalena valley | Cundinamarca -Ricaurte | Loc. 6 | 5.48 | 51.51 | 6.51 | 29.78 |
| ERR17368056 | FVP 069 | -74.664205 | 4.338407 | Upper Magdalena valley | Cundinamarca -Ricaurte | Loc. 6 | 5.23 | 51.49 | 6.5 | 29.79 |
| ERR17368008 | FVP 071 | -74.68107 | 6.059372 | Middle Magdalena valley | Antioquia - Puerto Nare | Loc. 7 | 9.53 | 62.07 | 6.77 | 30.06 |
| ERR17368012 | FVP 072 | -74.676063 | 6.059274 | Middle Magdalena valley | Antioquia - Puerto Nare | Loc. 7 | 9.53 | 62.07 | 6.77 | 30.06 |
| ERR17368015 | FVP 073 | -74.673881 | 6.063413 | Middle Magdalena valley | Antioquia - Puerto Nare | Loc. 7 | 9.25 | 60.32 | 6.82 | 30.08 |
| ERR17368017 | FVP 074 | -74.673874 | 6.06753 | Middle Magdalena valley | Antioquia - Puerto Nare | Loc. 7 | 9.33 | 61.72 | 6.85 | 30.08 |
| ERR17368020 | FVP 075 | -74.645919 | 6.014991 | Middle Magdalena valley | Antioquia - Puerto Nare | Loc. 7 | 8.4 | 50.38 | 7.32 | 30.09 |
| ERR17368023 | FVP 076 | -74.640709 | 6.025692 | Middle Magdalena valley | Antioquia - Puerto Nare | Loc. 7 | 8.21 | 50.6 | 7.22 | 30.1 |
| ERR17368024 | FVP 077 | -74.640998 | 6.028769 | Middle Magdalena valley | Antioquia - Puerto Nare | Loc. 7 | 8.21 | 50.6 | 7.22 | 30.1 |
| ERR17368042 | FVP 079 | -74.685739 | 6.04398 | Middle Magdalena valley | Antioquia - Puerto Nare | Loc. 7 | 9.62 | 60.85 | 6.7 | 30.07 |
| ERR17368043 | FVP 080 | -74.686947 | 6.04266 | Middle Magdalena valley | Antioquia - Puerto Nare | Loc. 7 | 9.62 | 60.85 | 6.7 | 30.07 |
| ERR17367862 | FVP 081 | -73.524777 | 4.292104 | Orinoquía | Meta - Cumaral | Loc. 8 | 6.64 | 38.92 | 7.63 | 29.9 |
| ERR17367864 | FVP 083 | -73.529952 | 4.271534 | Orinoquía | Meta - Cumaral | Loc. 8 | 6.63 | 38.95 | 7.59 | 29.9 |
| ERR17367866 | FVP 085 | -73.577325 | 4.222314 | Orinoquía | Meta - Restrepo | Loc. 8 | 7.73 | 45.09 | 7.16 | 29.92 |
| ERR17368131 | FVP 086 | -73.519649 | 4.20375 | Orinoquía | Meta - Restrepo | Loc. 8 | 6.05 | 36.96 | 7.93 | 29.95 |
| ERR17367877 | FVP 089 | -73.496284 | 4.187723 | Orinoquía | Meta - Restrepo | Loc. 8 | 5.43 | 34.16 | 8.32 | 29.96 |
| ERR17367879 | FVP 090 | -73.493028 | 4.169918 | Orinoquía | Meta - Restrepo | Loc. 8 | 5.25 | 33.46 | 8.31 | 29.97 |
| ERR17367881 | FVP 092 | -73.405523 | 4.172362 | Orinoquía | Meta - Restrepo | Loc. 8 | 4.39 | 30.06 | 9.06 | 29.99 |
| ERR17367769 | FVP 100 | -71.593036 | 5.415307 | Orinoquía | Casanare - Trinidad | Loc. 9 | 1.11 | 13.18 | 10.69 | 30.05 |
| ERR17367771 | FVP 102 | -71.595573 | 5.410248 | Orinoquía | Casanare - Trinidad | Loc. 9 | 1.11 | 13.18 | 10.69 | 30.05 |
| ERR17367773 | FVP 103 | -71.599064 | 5.417822 | Orinoquía | Casanare - Trinidad | Loc. 9 | 1.11 | 13.13 | 10.69 | 30.05 |
| ERR17367775 | FVP 104 | -71.560128 | 5.353909 | Orinoquía | Casanare - Trinidad | Loc. 9 | 1.19 | 13.22 | 10.81 | 30.05 |
| ERR17367778 | FVP 105 | -71.556227 | 5.357219 | Orinoquía | Casanare - Trinidad | Loc. 9 | 1.2 | 13.18 | 10.78 | 30.05 |
| ERR17367780 | FVP 106 | -71.5477217 | 5.35670167 | Orinoquía | Casanare - Trinidad | Loc. 9 | 1.21 | 13.14 | 10.78 | 30.05 |
| ERR17367782 | FVP 107 | -71.595536 | 5.387366 | Orinoquía | Casanare - Trinidad | Loc. 9 | 1.13 | 13.3 | 10.69 | 30.05 |
| ERR17367784 | FVP 108 | -71.600214 | 5.4223 | Orinoquía | Casanare - Trinidad | Loc. 9 | 1.1 | 13.22 | 10.55 | 30.05 |
| ERR17367785 | FVP 109 | -71.782919 | 5.257236 | Orinoquía | Casanare - San Luis de Palenque | Loc. 9 | 1.09 | 16.03 | 11.24 | 30.07 |
| ERR17367788 | FVP 111 | -71.789479 | 5.257367 | Orinoquía | Casanare - San Luis de Palenque | Loc. 9 | 1.09 | 16.15 | 11.29 | 30.07 |
| ERR17368072 | FVP 120 | -67.709426 | 6.263111 | Orinoquía | Vichada - Puerto Carreño | Loc. 12 | 0.98 | 7.16 | 12.67 | 30.15 |
| ERR17368074 | FVP 121 | -67.709988 | 6.286265 | Orinoquía | Vichada - Puerto Carreño | Loc. 12 | 0.93 | 6.88 | 12.84 | 30.16 |
| ERR17368076 | FVP 122 | -67.706522 | 6.28656 | Orinoquía | Vichada - Puerto Carreño | Loc. 12 | 0.93 | 6.87 | 12.71 | 30.16 |
| ERR17368077 | FVP 123 | -67.703155 | 6.286605 | Orinoquía | Vichada - Puerto Carreño | Loc. 12 | 0.93 | 6.87 | 12.71 | 30.16 |
| ERR17368078 | FVP 124 | -67.699405 | 6.289284 | Orinoquía | Vichada - Puerto Carreño | Loc. 12 | 0.93 | 6.86 | 12.76 | 30.16 |
| ERR17367737 | FVP 126 | -75.724003 | 6.508761 | Cauca valley - north | Antioquia - Sopetrán | Loc. 10 | 5.48 | 45.58 | 2.27 | 29.58 |
| ERR17367743 | FVP 127 | -75.722914 | 6.509631 | Cauca valley - north | Antioquia - Sopetrán | Loc. 10 | 5.48 | 45.58 | 2.27 | 29.58 |
| ERR17367750 | FVP 128 | -75.724065 | 6.510311 | Cauca valley - north | Antioquia - Sopetrán | Loc. 10 | 5.48 | 45.58 | 2.27 | 29.58 |
| ERR17367753 | FVP 129 | -75.728346 | 6.5022 | Cauca valley - north | Antioquia - Sopetrán | Loc. 10 | 5.24 | 43.75 | 2.25 | 29.67 |
| ERR17367757 | FVP 130 | -75.783037 | 6.554781 | Cauca valley - north | Antioquia - Olaya | Loc. 10 | 5.01 | 55.18 | 2.09 | 29.98 |
| ERR17367761 | FVP 131 | -75.785803 | 6.554221 | Cauca valley - north | Antioquia - Olaya | Loc. 10 | 5 | 45.07 | 2.15 | 30 |
| ERR17367764 | FVP 133 | -75.800714 | 6.473908 | Cauca valley - north | Antioquia - Sopetrán | Loc. 10 | 4.87 | 42.75 | 2.32 | 29.95 |
| ERR17367767 | FVP 134 | -75.805018 | 6.473982 | Cauca valley - north | Antioquia - Sopetrán | Loc. 10 | 4.87 | 42.75 | 2.32 | 29.95 |
| ERR17368057 | FVP 135 | -75.886821 | 4.753719 | Cauca valley - south | Cauca valley - Cartago | Loc. 11 | 10.03 | 47.63 | 1.55 | 29.53 |
| ERR17368059 | FVP 137 | -75.925719 | 4.756875 | Cauca valley - south | Cauca valley - Cartago | Loc. 11 | 8.65 | 50.22 | 1.72 | 29.54 |
| ERR17368061 | FVP 138 | -75.92753 | 4.763303 | Cauca valley - south | Cauca valley - Cartago | Loc. 11 | 8.75 | 41.67 | 1.42 | 29.55 |
| ERR17368062 | FVP 140 | -75.951001 | 4.808435 | Cauca valley - south | Cauca valley - Ansermanuevo | Loc. 11 | 9.43 | 54.12 | 1.79 | 29.55 |
| ERR17368064 | FVP 141 | -76.071344 | 4.317435 | Cauca valley - south | Cauca valley - Zarzal | Loc. 11 | 8 | 45.22 | 1.79 | 29.55 |
| ERR17368065 | FVP 142 | -76.064679 | 4.330641 | Cauca valley - south | Cauca valley - Zarzal | Loc. 11 | 8.12 | 45.9 | 1.71 | 29.55 |
| ERR17368067 | FVP 143 | -76.047387 | 4.387796 | Cauca valley - south | Cauca valley - Zarzal | Loc. 11 | 7.86 | 45.06 | 1.53 | 29.53 |
| ERR17368068 | FVP 144 | -75.953602 | 4.61077 | Cauca valley - south | Cauca valley - Cartago | Loc. 11 | 7.54 | 25.31 | 1.58 | 29.53 |
| ERR17368223 | FVP 145 | -75.908672 | 4.751841 | Cauca valley - south | Cauca valley - Cartago | Loc. 11 | 9.06 | 52.26 | 1.65 | 29.54 |
| ERR17368071 | FVP 146 | -75.921 | 4.759553 | Cauca valley - south | Cauca valley - Cartago | Loc. 11 | 8.93 | 42.55 | 1.42 | 29.55 |

**Table S2**. Description of the twelve sampling localities in Colombia.

**Caribbean Region**

**Locality 1 / Piojó (Atlántico):** Located in lowland dry forest patches in the Caribbean region of Colombia, within the Atlántico department. Forests here show varying degrees of degradation due to human activities, but many conserved forest patches remain. Individuals were collected both from forest patches near streams and from open areas away from streams.

**Locality 2 / Luriza (Atlántico):** Situated in lowland dry forest patches within the Atlántico department, near **Locality 1**. Trees were mainly collected from relatively conserved forest patches along seasonal water corridors. These corridors dry up after rainfall ceases and are mostly washed out during the dry season due to severe drought.

**Locality 3 / Minca (Magdalena):** Located in the piedmont of the Sierra Nevada de Santa Marta, a coastal mountain range in the Magdalena department. This site lies at the transition between dry and montane forests, resulting in mixed vegetation. The locality includes both conserved and degraded patches; most samples came from semi-conserved forests.

**Locality 4 / Mingeo (Guajira):** Found in lowland dry forest patches near the coast in the Guajira department. Most trees were collected in degraded forest areas, as they were absent from the most conserved patches.

**Locality 5 / La Jagua de Ibirico (Cesar):** Located in lowland to piedmont areas of the eastern Andes, within the Cesar department. Vegetation is a mixture of montane forests and lowland dry forests. The sampled area includes both conserved and degraded patches, as well as cattle ranches and cultivated areas. Trees were collected along streams and in open areas.

**Orinoquía**

**Locality 8 / Restrepo and Cumaral (Meta):** Situated at the interface between dry forests at the piedmont of the eastern Andes and the Orinoquía. The landscape is highly degraded due to cattle ranching, and most trees occur in human-modified and open areas or near to rivers.

**Locality 9 / Trinidad and San Luis de Palenque (Casanare):** Located in the Casanare savannas, which, like most in Colombia, have been heavily modified by human activities. Trees were mainly found in agricultural landscapes and cattle ranches.

**Locality 12 / Puerto Carreño (Vichada):** Located in the Vichada department near the border with Venezuela. Trees were scarce and absent from areas affected by seasonal fires. Samples came from a cattle ranch near the Meta River, with only five trees collected.

**Cauca valley**

**Locality 10 / Sopetrán and Olaya - Cauca Valley North (Antioquia):** Situated in the lowland dry forests of the Cauca Valley, between the Central and Western Andes, in Antioquia. The landscape includes both conserved and degraded forest patches. Samples were taken from both, but most trees were found in semi-open and open areas compared with the better-preserved dry forests of the Caribbean region.

**Locality 11 / Cartago, Ansermanuevo and Zarzal - Cauca Valley South (Valle del Cauca):** Located in the middle to southern Cauca Valley. This area is heavily degraded, with no conserved dry forest patches remaining. Trees were mostly collected from degraded landscapes, including semi-urban areas and cattle ranches.

**Magdalena valley**

**Locality 7 / Puerto Nare - Middle Magdalena Valley (Antioquia):** Located in the Middle Magdalena Valley, a lowland valley between the Eastern and Central Andes. The dry forests here are also heavily degraded. Samples were taken from open areas within cattle ranches, likely remnants of a once more extensive forest.

**Locality 6 / Nilo and Ricaurte - Upper Magdalena Valley (Cundinamarca):** Situated in the Upper Magdalena Valley. The landscape includes both conserved and degraded patches, but individuals were absent from the most conserved areas. Most trees were found in degraded landscapes, near streams, and in open modified areas, probably remnants of a formerly larger population.

**References**

Luu, K., Bazin, E., & Blum, M. G. B. (2017). pcadapt: An R package to perform genome scans for selection based on principal component analysis. *Molecular Ecology Resources*, *17*(1), 67-77.

Privé, F., Luu, K., Vilhjálmsson, B. J., & Blum, M. G. B. (2020). Performing highly efficient genome scans for local adaptation with R package pcadapt version 4. *Molecular Biology and Evolution*, *37*(7), 2153-2154.

Storey, J. D., & Tibshirani, R. (2003). Statistical significance for genomewide studies. *Proceedings of the National Academy of Sciences*, *100*(16), 9440-9445.

Weir, B. S., & Cockerham, C. C. (1984). Estimating F-statistics for the analysis of population structure. *Evolution*, *38*(6), 1358.
